# T-cell receptor-based therapy: an innovative therapeutic approach for solid tumors

**DOI:** 10.1186/s13045-021-01115-0

**Published:** 2021-06-30

**Authors:** Apostolia-Maria Tsimberidou, Karlyle Van Morris, Henry Hiep Vo, Stephen Eck, Yu-Feng Lin, Jorge Mauricio Rivas, Borje S. Andersson

**Affiliations:** 1 Department of Investigational Cancer Therapeutics, Unit 455, Phase I Clinical Trials Program, The University of Texas MD Anderson Cancer Center, 1515 Holcombe Blvd, Houston, TX 77030 USA; 2 Department of Gastrointestinal Medical Oncology, The University of Texas MD Anderson Cancer Center, 1515 Holcombe Blvd, Houston, TX 77030 USA; 3 MacroGenics, Inc., 9704 Medical Center Drive, Rockville, MD 20850 USA; 4 Immatics US, Inc., 2201 Holcombe Blvd., Suite 205, Houston, TX 77030 USA; 5 Department of Stem Cell Transplantation, The University of Texas MD Anderson Cancer Center, 1515 Holcombe Blvd, Houston, TX 77030 USA

**Keywords:** Adoptive T-cell receptor-based therapy, Human leukocyte antigen typing, Biomarker screening, Lymphodepletion, Clinical trials, Solid tumors

## Abstract

T-cell receptor (TCR)-based adoptive therapy employs genetically modified lymphocytes that are directed against specific tumor markers. This therapeutic modality requires a structured and integrated process that involves patient screening (e.g., for HLA-A*02:01 and specific tumor targets), leukapheresis, generation of transduced TCR product, lymphodepletion, and infusion of the TCR-based adoptive therapy. In this review, we summarize the current technology and early clinical development of TCR-based therapy in patients with solid tumors. The challenges of TCR-based therapy include those associated with TCR product manufacturing, patient selection, and preparation with lymphodepletion. Overcoming these challenges, and those posed by the immunosuppressive microenvironment, as well as developing next-generation strategies is essential to improving the efficacy and safety of TCR-based therapies. Optimization of technology to generate TCR product, treatment administration, and patient monitoring for adverse events is needed. The implementation of novel TCR strategies will require expansion of the TCR approach to patients with HLA haplotypes beyond HLA-A*02:01 and the discovery of novel tumor markers that are expressed in more patients and tumor types. Ongoing clinical trials will determine the ultimate role of TCR-based therapy in patients with solid tumors.

## Background

Immunotherapy has significantly improved the outcomes of patients with selected tumor types. Adoptive cell therapy (ACT), which uses genetically engineered human lymphocytes, is increasingly being investigated in patients with hematologic malignancies and solid tumors.

**Fig. 1:**
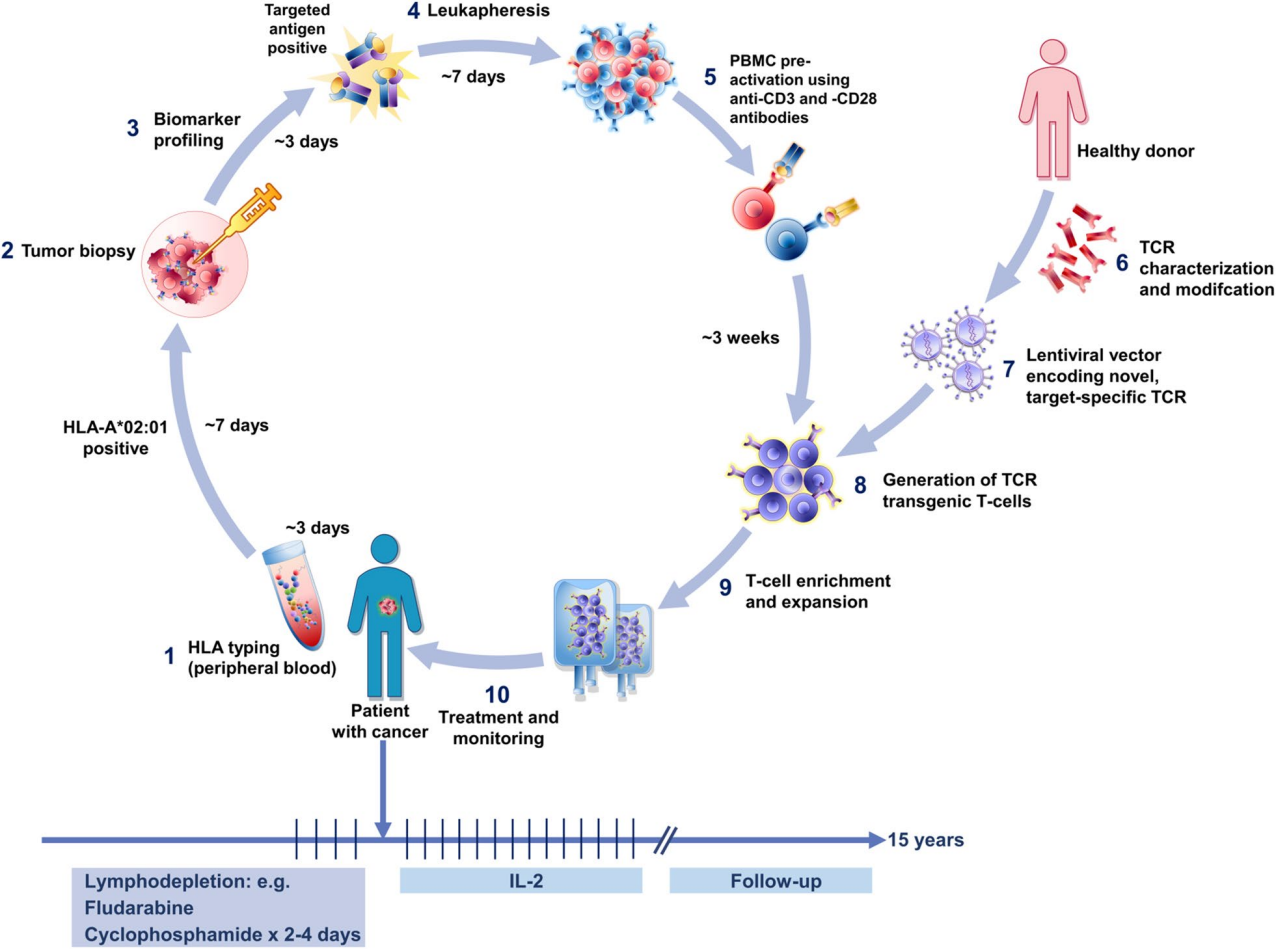
Schematic view of TCR-based adoptive T-cell therapy. (1) Patient’s screening starts with HLA typing. If HLA is A*02:01 type, a tumor biopsy is performed (2) to screen the tumor tissue for the expression of the targeted antigen (3), followed by leukapheresis (4). PBMCs from patient leukapheresis are isolated and pre-activated using anti-CD3 and -CD28 antibodies (5). A target-specific TCR is isolated from a healthy donor, characterized, and modified (6). A lentiviral vector is constructed and used to transfer the target-specific TCR in the T-cells (7). The activated PBMCs are transduced with a lentiviral vector encoding the target-specific TCR (8). Transduced T-cells are expanded to large numbers in 3–5 days and are frozen (9). Upon completion of the release testing, the T-cells are ready to be infused (10). Patients are typically treated with lymphodepletion, followed by T-cell product infusion, followed by low-dose interleukin 2. Patients are monitored for as long as 15 years to observe for delayed adverse events following exposure to the investigational gene therapy product

**Fig. 2:**
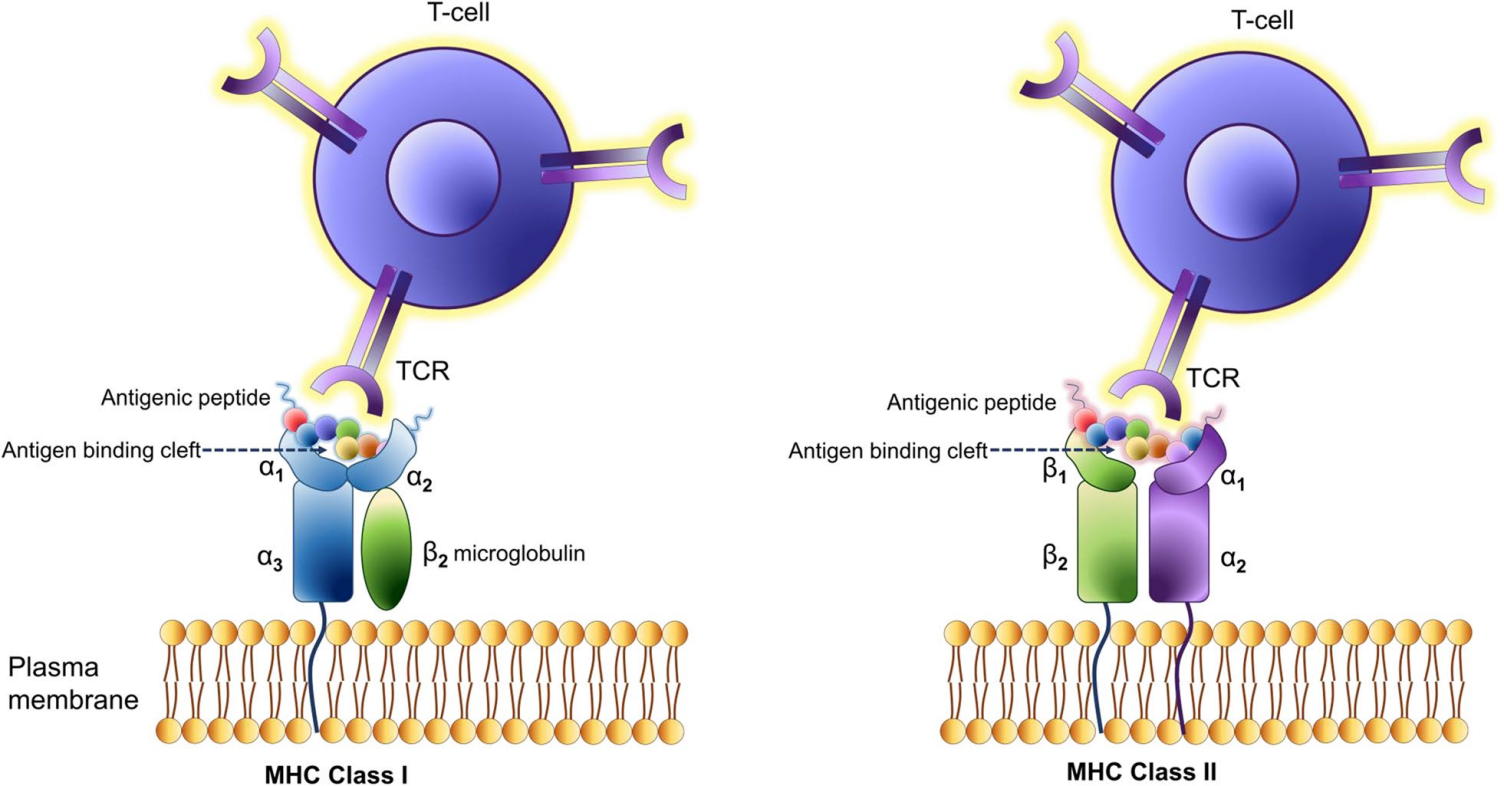
Schematic view of MHC class I and MHC class II molecules. MHC class I and class II molecules have high levels of polymorphism; a similar three-dimensional structure; a genetic location within one locus; and a similar function in presenting peptides to the immune system. MHC class I molecules present peptides at the cell surface to CD8 + T-cells, whereas MHC class II molecules present peptides to CD4 + T-cells that are derived from proteins degraded in the endocytic pathway. MHC class II molecules are primarily expressed by professional antigen-presenting cells (APCs), such as dendritic cells, macrophages, and B cells, and are conditionally expressed by other cell types. The transmembrane α- and β-chains of MHC class II molecules are assembled in the ER and associate with the invariant chain (Ii). The resulting Ii-MHC class II complex is transported to a late endosomal compartment termed the MHC class II compartment (MIIC). Here, the variant chain is digested, leaving a residual class II-associated Ii peptide (CLIP) in the peptide-binding groove of the MHC class II heterodimer. In the MIIC, MHC class II molecules require the chaperone HLA-DM to facilitate the exchange of the CLIP fragment for a specific peptide derived from a protein degraded in the endosomal pathway. MHC class II molecules are then transported to the plasma membrane to present their peptide cargo to CD4 + T-cells. In B cells, a modifier of HLA-DM is expressed called HLA-DO, and this protein associates with HLA-DM and restricts HLA-DM activity to more acidic compartments, thus modulating peptide binding to MHC class II molecules

ACT, through the infusion of ex vivo-activated autologous or allogeneic T-cells, with or without other agents that combat T-cell inhibition in the tumor microenvironment, can overcome the limitations of some current immunotherapies. Extensive libraries of T-cell epitopes are being constructed to address the needs of as many patients with cancer as possible with increasingly customized approaches [1]. Two general approaches to ACT are being developed. Chimeric antigen receptor (CAR) technology (now available in marketed products) uses an artificial receptor introduced into the immune effector cells to recognize tumor cell surface proteins. In contrast, T-cell receptor (TCR)-engineered effector cells use a naturally occurring (or minimally modified) TCR to develop T-cell-based adoptive T-cell therapy (Fig. 1). This approach has been selected for its ability to recognize tumor-specific epitopes presented by the major histocompatibility complex (MHC) molecules on the tumor cell surface (Fig. 2). The latter strategy has a potentially broader applicability, as there are far more tumor-specific sequences within a cell and presented in the MHC than there are tumor-specific proteins on the surface. These intracellular cancer targets are only accessible by TCR-based approaches and not by CAR-based approaches. ACT can in principle utilize a variety of effector cells, but it is most commonly based on T-cells or natural killer (NK) cells derived from the patient and genetically modified. Regardless of the approach, several clinical trials have demonstrated remarkable responses to ACT [2].

In this review, we focus on TCR-based therapy, specifically its technical development and clinical implementation including candidate TCR identification/characterization, target antigen screening, individual patient product manufacturing, patient lymphodepletion, and subsequent treatment. This review summarizes the lines of investigation and products that are currently being developed by biotechnology companies to treat solid tumors using TCR-based therapies. Comparisons of CAR- and TCR-based therapies and the functions of tumor targets used for TCR-based therapy and tumor types associated with their overexpression are also reviewed.

### Mechanisms of action

Most immunotherapies fail because they are unable to deliver an effective pool of anti-tumor effector cells and/or because the effector cells mobilized are inhibited by tumor-associated factors. TCR-based ACT overcomes the first of these barriers by the ex vivo manufacture of up to billions of activated lymphocytes with known selectivity and potency. The majority of TCR structures are heterodimers comprised of α- and β-chains that are covalently linked via a disulfide bond between the conserved cysteine residues located within the constant region of each chain [3]. Neither TCR chain has intrinsic signaling capacity, and activation requires interaction between the TCR and other accessory signaling molecules. A non‐covalent oligomeric complex comprised of TCR and CD3 signaling molecules (CD3ζ, CD3δε, and CD3γε) initiates signaling activity on binding a cognate peptide MHC complex on the target cell and enables antigen-specific tumor cell lysis [3, 4].

Class I MHC complexes present cleaved peptides generated primarily from intracellular proteins [5] and thereby have the potential to present fragments of normal proteins, tumor-specific mutated proteins, or aberrantly transcribed cancer-associated differentiation antigens [e.g., melanoma antigen gene (MAGE), New York esophageal squamous cell carcinoma (NY-ESO)] [6–8]. For any given peptide-MHC target selected for its cancer specificity, multiple TCRs can be identified and an optimal TCR selected. Having done so, it is not as easy to identify all the other peptide MHC complex in which the selected TCR also binds. The ability of the newly introduced therapeutic TCRs to recognize more than one peptide-MHC complex and even multiple peptides within a specified MHC can potentially lead to “off-target” and “off-tumor” effects. The diversity of peptides potentially recognized by one TCR and the possibility of normal tissue injury is partly, but not completely, addressed by pre-clinical screening of candidate TCRs [9–11] (Fig. 1).

TCRs expressed by CD8 + T-cells recognize a common peptide antigen consisting of 8‐11 amino acid residues in complex with MHC class I molecules [12]. Other CD4 or CD8 co-receptors expressed by T-cells bind to the conserved motifs in the MHC molecule and stabilize TCR/MHC interactions without direct interaction with the presented peptide [13, 14]. The repertoire of T-cells that interact with tumor-associated antigens is vast, although many TCR-peptide MHC interactions are of low affinity [15]. TCRs can respond to a low density of molecules on a target cell. While the optimum density is unknown, TCRs have been shown to induce antigen‐specific cytokine release in response to as few as one peptide/MHC complex [16, 17].

The strength of the TCR affinity for peptide and MHC complexes determines the activation of lymphocytes. It has been shown that the immune response to foreign antigens is dominated by CD8(+) T-cells with higher peptide reactivity, which has implications for T-cell repertoire diversity and autoimmunity [18].

There are two general approaches to ACT. Historically, therapeutic lymphocytes were produced by the ex vivo expansion of autologous T-cells harvested from the tumor (e.g., tumor-infiltrating lymphocytes [TILs]) or from peripheral blood mononuclear cells (PBMCs). This approach yields a T-cell product that reflects the naturally occurring repertoire of TCRs and is infused as a largely unmodified product, although it is recognized that the ex vivo culture conditions may enhance its performance. The principal limitation of this approach is that it is unclear whether the TCRs will be able to efficiently kill tumor cells, as they may be of low affinity or have other unfavorable biochemical properties. A more recently developed approach features the ex vivo expansion of anti-tumor T lymphocytes after they have been genetically modified by the ex vivo insertion of genes encoding carefully selected TCRs of known specificity and affinity [19]. In the latter case, autologous peripheral blood lymphocytes are genetically engineered to express a novel TCR (or CAR) that recognizes specific tumor antigens [20]. The selection of and design of the receptor (if modified), as well as the vector methodology, has been greatly refined with successive generations of experimental products.

For the development of safe and effective TCR-based adoptive therapy, the selection of the antigen and the cognate TCR are of vital importance. Target antigens should be selectively expressed in tumors and not (or only at very low levels) expressed in normal tissues. Consequently, a specific and selective TCR with sufficient target affinity and minimal cross-reactivity against other peptides is needed [21]. In addition, an effective and robust T-cell transduction and expansion process must be developed that allows the reliable delivery of a potent and safe immunotherapy product to the patient. The transduction efficiency is of paramount importance, as there is significant patient-to-patient variation in the number of T-cells collected for manufacture of the ACT product.

### Tumor characteristics

The tumor mutational burden is a rough indicator of the likelihood of a tumor-specific somatic mutation leading to immune-mediated tumor eradication, but this often fails to occur even in tumors with Mis-Match Repair deficiency (MMR deficiency) or high microsatellite instability (MSI) both of which can lead to 10 to 100 times as many somatic mutations. Immune check points account for part of the lack of spontaneous responses to such neoantigens, as revealed by the increased clinical responses seen when immune check point inhibitors are used as therapeutic agents. However, neoantigen quantity appears to be less important than neoantigen quality in determining response to immunotherapies. Specifically, the efficiency of neoantigen presentation to T-cells determines the efficiency of T-cell activation. Additionally, approximately 40–90% of human tumors are MHC class I deficient, a feature associated with an invasive, metastatic tumor phenotype [22]. MHC-I-positive tumor clones are highly immunogenic, whereas MHC-I-negative variants have low immunogenicity [23]. This raises the unfortunate possibility of selectively killing the MHC-positive cells while leaving intact the MHC-negative tumor cells.

Tumor neoantigens (derived from tumor somatic mutations or aberrant mRNA processing) are peptides that are absent from normal human tissues and potentially recognized by TCRs if presented by MHC molecules [24–27]. Neoantigens thus are important targets in tumor-specific T-cell-mediated antitumor immune response and other cancer immunotherapies [28]. Sources of neoantigens include somatic gene mutations, variant RNA splicing, and derivatives of embryo-fetal proteins (not expressed in normal adult tissues) [28].

### Optimization of TCR-based therapy

TCRs must be selected on the basis of being unlikely to have cross-reactivity with structurally similar peptide antigens expressed by normal tissue [11]. While the TCR must have high specificity for the appropriate MHC-peptide complex (currently most typically HLA-A*02:01), it does not necessarily have to be isolated from an individual with the same MHC profile as the intended patient. The nature of the interaction between TCRs and their ligands, the strength of this interaction, and the environment (e.g., including, but not limited to, presence of PD-1-PD-L1 interactions) determine the response of the T-cell. Challenges with heterotopic expression of an introduced novel TCR includes cross pairing of α- and β-TCR chains from the introduced TCR with those of the endogenous TCR. Such cross-pairing carries the potential risk of mixed dimer formation giving rise to a new TCRs with unpredictable specificity. In addition, there is competition for cellular resources when a new TCR is introduced. Unlike an introduced CAR, the newly introduced “therapeutic” TCRs compete with the endogenous TCR for the accessory CD3 signaling proteins. The αβ TCR proteins associate with the CD3γε–CD3δε–CD3ζζ signaling hexamer. This octameric complex determines T-cell activation and responses to antigens. The introduction of new α- and β-TCR proteins, without the silencing of expression of the endogenous α- and β-TCR proteins, could disrupt the stoichiometry required for efficient assembly of an active TCR-CD3 complex.

#### Lymphodepletion regimen

The rationale for including lymphodepleting chemotherapy prior to infusion of T-cell products is based on the following three assumptions: (a) genetically modified T-cells risk being recognized as non-self; therefore, eradication of the preexisting immune reactive cells will promote the survival of the transfused T-cells; (b) lymphodepletion imposes normal organ stress to facilitate release of interleukins and other growth stimulatory factors to promote the expansion and proliferation of the transfused T-cells; (c) if fludarabine is included in the regimen, it appears that it favors the interaction of antigen-presenting cells with T-cells, leading to enhanced T-cell response.

There is no consensus as to what is the optimal lymphodepletion regimen at this time and randomized studies with different schedules have not been conducted. As the engraftment and persistence of transferred T-cells depends on the lymphodepletion regimen [29–31], published studies have used radiation therapy (XRT)-based lymphodepletion regimens with XRT doses. In a study in melanoma, non-myeloablative chemotherapy was combined with low-dose (2 GY) or high-dose (12 GY) total body irradiation (TBI) [30, 32]. Although high-dose TBI had significant benefit, it was also associated with risks, including severe and prolonged myelosuppression and development of secondary tumors. Additionally, in patients who underwent allogeneic stem cell transplantation, emerging data suggest that chemotherapy alone is as effective as chemotherapy plus TBI, but not associated with the long-term complications of TBI. Therefore, borrowing strength from these data, many groups elected to use chemotherapy alone (without TBI) as the basis for non-myeloablative lymphodepletion. Fludarabine and cyclophosphamide (FC) combination regimens have become somewhat of a standard for TIL trials and in ACT trials using TCR-engineered T-cells, although there is wide variation in the doses of fludarabine and cyclophosphamide used. Remarkable clinical effects were reported from trials using this regimen, but it is also associated with substantial toxicities [33, 34]. For therapy with autologous ex vivo-expanded non-engineered T-cells, as in the ACTolog IMA101-101 trial [1], no standard regimen has been established and no major differences in clinical responses have been reported/observed using different regimens. The lack of discernable differences, however, could be explained by the small numbers of patients with a variety of different heavily pre-treated malignancies in those studies, which would easily obfuscate the contribution of an optimized lymphodepletion (LD) regimen to treatment outcome.

Among the LD regimens used at The University of Texas MD Anderson Cancer Center, the modified FC (mFC) LD regimen used in the IMA101-101 trial [1] is a version of the FC regimen that is expected to lead to lymphodepletion comparable to that of the “standard” FC but with a more favorable safety profile. This mFC is building on the mechanistic model cell line studies of Yamauchi et al. [35] and Valdez and Andersson [36]. In the design of this program, it was hypothesized that FC would benefit from being optimized for both the timing and sequencing of the two drugs to achieve synergistic cell kill/lymphodepletion but without excessive normal organ toxicity. Further, fludarabine has a very long half-life, which raises a need for at least two to three rest days after completion of the chemotherapy so that the infused T-cells will not be inadvertently killed off by fludarabine still in the circulation, something found detrimental to patients receiving a cord-blood transplant after analogous conditioning therapy. Additionally, any renal impairment that would further delay fludarabine clearance needs to be taken into consideration [37, 38]. Finally, it has been suggested that FC may alter antigen presentation, improving the interaction between the tumor antigens and the transferred T-cells, further strengthening the case for optimizing the dose and timing of the lymphodepletion regimen [29].

In reference to using XRT/TBI for lymphodepletion and given the previous observations of the benefit of TBI, one can speculate that incorporation of stereotactic XRT to treat suitable tumors would not only allow for intensive radiation to local tumor sites, but it might also improve T-cell homing and the antitumor efficacy of the T-cell product. Aside from delivering a very high, targeted XRT dose, stereotactic XRT can be administered over just a few days, similar to the aforementioned reported TBI dose(s) [30, 32] that were found to elicit excellent antitumor responses when followed by T-cell therapy. Thus, the benefit of a highly cytoreductive, focused XRT program could be combined with the benefits of the T-cell program, analogous to the situation with standard-dose FC related above.

A different approach has been proposed by June and colleagues, who recently suggested replacing standard chemotherapy agents for lymphodepletion with intratumoral injections of adenovirus to facilitate T-cell homing and expansion in selected tumor types expressing mesothelin (personal communication, Dr. Carl June, October 2020).

### Cells used for TCR

#### αβT-cells and γδT-cells

The dynamic responses of T-cells to pathogens and tumor cells are mediated through the diversity of their individual TCRs. The majority of TCRs expressed by CD8 + T-cells are composed of an α- and a β-chain (αβT-cells). Activation of αβT-cells depends on specific tumor antigen expression, derived from proteins expressed in cancer cells and presented in a defined HLA molecule [39]. A small subset of CD8 + T-cells (1–10%) express TCRs composed of γ- and δ-chains (γδT-cells) [40, 41]. γδT-cells are distinct from αβT-cells in antigen recognition, activation, development of an antigen-specific repertoire, and effector function [42, 43]. The precise mechanisms by which γδT-cells function are unclear but involve production of interferon-γ (IFN‐γ) and tumor necrosis factor (TNF). Release of IL-17 by γδT-cells in concert with chemotherapeutic drugs has been reported to induce immunogenic cell death [42, 44].

Most cellular engineering approaches have been applied to αβT-cells derived from peripheral blood [45–47]. The transfer of a new α’β’ TCR gene construct into an αβT-cell is associated with the risk of TCR chain mis-pairing (e.g., α’β or αβ’ TCRs), unless the endogenous α- and β-chains are suppressed [48]. Mis-pairing may lead to self-reactive TCR clone generation and off-target toxicity [49]. Using murine constant regions or altering the arrangement of cysteines in the transferred TCRs may decrease mis-pairing [50]. γδT-cells exhibit innate and adaptive immune properties and can be used as the substrate for insertion of αβ T chains [41]. The use of γδT-cells for TCR engineering may overcome the mis-pairing issue because the endogenous γ and δ TCR chains cannot mis-pair with transfected α or β proteins. γδT-cells can be modified using engineering techniques similar to those used for modifying αβT-cells. However, the γδT-cells may be more effective owing to their innate-like tumor recognition and killing [45]. Engineered γδT-cells were shown to produce more IFN-γ and TNF-α than CD8 + αβT-cells expressing the same TCR and had equivalent cytotoxicity against autologous adenovirus-infected dendritic cells [51].

αβT-cell immune systems cannot be transferred between individuals unless all of the HLA molecules are precisely matched. Transferring γδT-cell immune systems between individuals may be less restricted and allow the use of γδT-cells from normal volunteers who would serve as “universal donors.”[42] The practical advantage is the avoidance of patient-specific leukapheresis to collect T-cells and patient-specific manufacturing using autologous cells. Allogeneic γδT-cells could, in principle, be an “off the shelf” product with one donor providing a T-cell product for multiple patients, decreasing cost and time significantly.

#### NK cells

NK cells may also be used in TCR-based therapy to overcome the challenge of mis-pairing [52]. NK cells are naturally cytotoxic against cancer and virus-infected cells and are not restricted by MHC [53–55]. Inserting TCR complexes into NK cell lines leads to the MHC-restricted, antigen-specific killing of tumor cells in vitro and in vivo [52]. NK cells genetically modified with TCRs have demonstrated the capability to recognize and kill tumor cells [56]. Clinical trials with allogeneic and autologous NK cell infusions demonstrated minimal side effects and encouraging antitumor responses [57]. Genetically modified NK cells targeting tumor-associated antigens through the expression of TCRs [58] have also shown encouraging results in clinical studies [59].

### Adjunctive therapy

Interleukin 2 (IL-2) has been widely used in immunotherapy trials and in ACT studies. IL-2 was first developed as single-agent therapy for metastatic melanoma, kidney cancer, and non-Hodgkin lymphoma, where it shows some benefit in eliciting anti-tumor immune responses (50% tumor reduction in 15–20% of patients), presumably by activating T lymphocytes [60, 61]. However, when high-dose IL-2 was administered together with TILs, objective tumor regression could be observed in 34% of patients who were refractory to single-agent IL-2 treatment [62]. High-dose (600,000 to 720,000 IU/kg every 8 h) and low-dose (0.5 to 2 × 10^6^/m^2^ per day) IL-2 have been widely applied in TIL and other ACT trials, and its administration is associated with increased T-cell persistence [63, 64]. However, treatment with high-dose IL-2 often results in life-threatening toxicities. In many trials, lymphostimulation with low-dose IL-2 is used to minimize IL-2-related toxicities while supporting long-term persistence of the T-cell transplant. The requirement of administering IL-2 after T-cell infusion in patients who participate in ACT trials may depend on the manufacturing system (with or without IL-2). It can be speculated that the effect of IL-2 during manufacturing T-cells could lead to cellular dependence on IL-2 after cell infusion. This could impact the in vivo expansion of infused cells driven by the administered IL-2.

### Comparison between TCR and CAR T-cell therapies

**Fig. 3:**
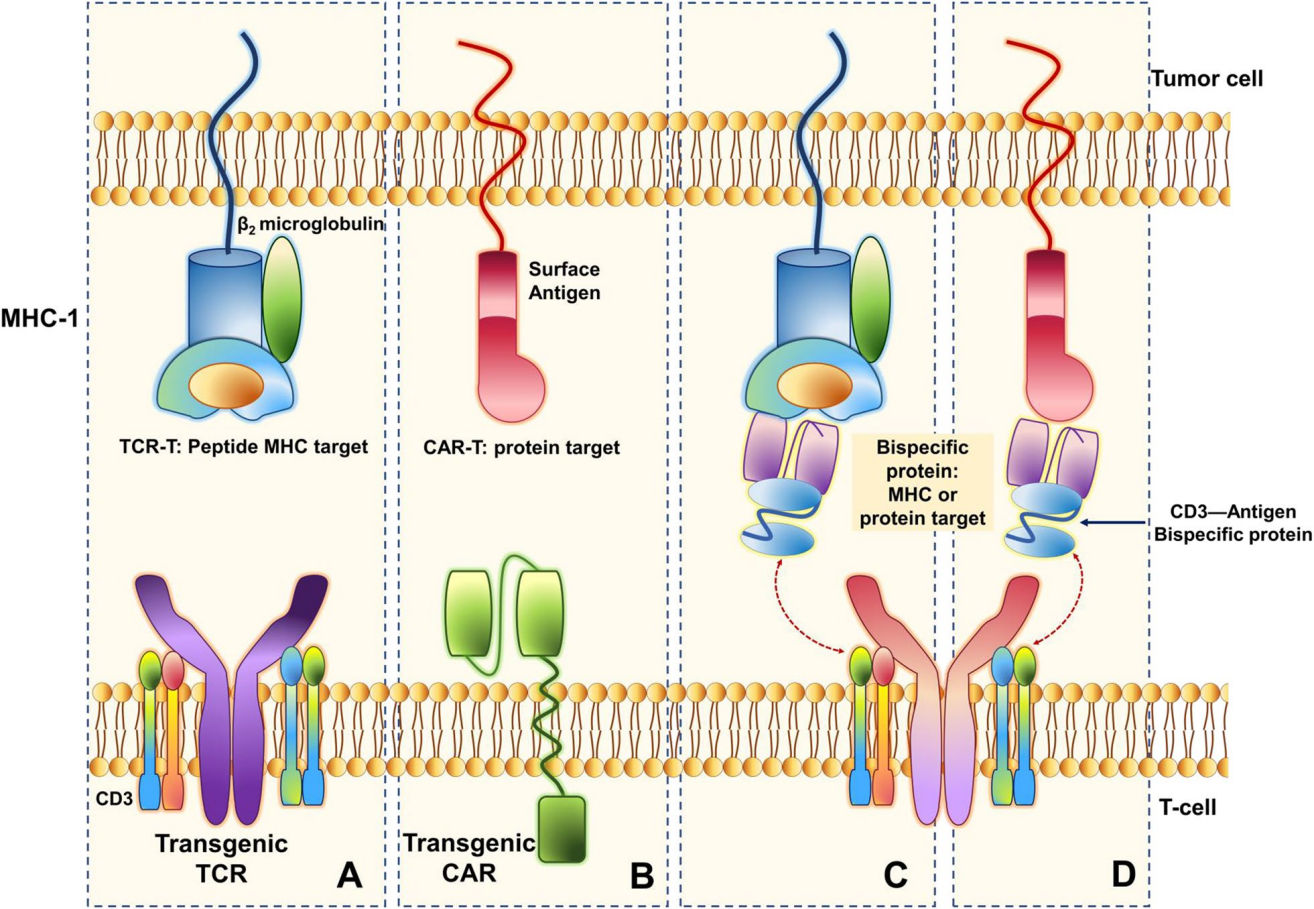
Schematic view of TCR and CAR structures. **a** TCR and CD3 molecules form a non-covalent TCR/CD3 receptor complex on the T-cell surface that recognizes and binds to an antigen peptide presented by MHC. **b** Transgenic CAR expressed on the surface of the T-cell recognizes a protein target (surface antigen) on the tumor cell **c**. A bispecific antibody (e.g., CD3 antigen bispecific protein) can bind to different antigens presented by MHC and/or **d**. A protein target on the surface of the tumor cell can be recognized by the TCR/CD3 complex

Understanding the differences between the CAR- and TCR-engineered T-cell receptor structures may aid in the appreciation of the associated functional differences (Fig. 3). Such distinctions account for specific treatment-associated toxicity profiles as well as provide context for expected responses. CAR T-cells were pioneered for B-cell leukemias and lymphomas and are less well developed for solid tumors. TCR T-cells may prove to be a more effective option for solid tumors where intracellular antigens presented in MHC (not recognizable by CAR T-cells) can differentiate cancer cells from normal tissues. A comparison between TCR T-cells and CAR T-cells is summarized in Table 1. Table 1 also includes CD3-directed bispecific antibodies and TCRs in the comparison. This promising class of drugs is engineered for dual binding to either MHC peptides or surface proteins and glycans and redirect endogenous T-cells to kill target cells leading to polyclonal expansion of T-cells.

**Table 1:** Comparison between TCR-T, CAR-T, and CD3-directed bispecific antibodies and TCRs

	Modified TCR expressed on T-cells, NK cells, and other cells	CAR expressed on T-cells, NK cells, and other cells	CD3-directed bispecific antibodies and TCRs
Constructs	Native or minimally engineered native TCR delivered via biologic vector	Artificial receptor complex delivered by a biologic vector	Antibody-like construct engineered for dual binding
Targets	MHC peptides derived from intracellular proteins	Surface proteins and glycans	Either MHC peptides or surface proteins and glycans
Manufacturing	Ex vivo gene transfer into autologous T-cells or NK cells, “personalized” for each patient	Ex vivo gene transfer into autologous T-cells or NK cells, “personalized” for each patient	“Off-the-shelf” conventional protein
Mechanism of action	Binds and kills target cells leading to limited clonal expansion of T-cells	Binds and kills target cells leading to extensive clonal expansion of T-cells	Redirects endogenous T-cells to bind and kill target cells leading to polyclonal expansion of T-cells
Dosing	Single or limited doses	Single or limited doses	Repetitive dosing
Availability	Experimental basis only	Experimental and commercially available products	Experimental and commercially available products
Unique facets	Small patient populations for any single construct	Limited number of suitable potential targets	Complex drug protein design needed to achieve optimal binding characteristics
Safety	Modest cytokine release syndrome due to limited proliferation	Extensive cytokine release syndrome due to extensive cell proliferation	Cytokine release syndrome easily managed by adjusting dose and infusion rate
Mechanism of resistance	Loss of target, loss of IFNγ signaling	Loss of target, loss of IFNγ signaling	Loss of target; loss of target fucosylation

*CAR* chimeric antigen receptor, *IFNγ* interferon gamma, *MHC* major histocompatibility complex, *NK* natural killer, *TCR* T-cell receptors. References [20, 108]

CARs structurally are composed of specifically engineered extracellular and intracellular components to mimic a true TCR, with each component critical to the function of the engineered anti-tumor CAR T-cell. An extracellular antibody-like domain is composed of a single-chain variable fragment (consisting of one variable heavy chain fragment fused to a variable light chain fragment) [65, 66] and serves to bind a specific epitope on a malignant cell surface protein and trigger intracellular signaling leading to T-cell activation, mediated by the intracellular T-cell-activating domain of the CAR (most commonly engineered as CD3ζ motifs) [67]. Potentiation of T-cell activation and survival of CAR T-cells are further enhanced by the addition of costimulatory domains to the intracellular portion of the CAR T-cell. Such domains (derived from CD28, 4-1BB, and ICOS [68] co-stimulatory molecules) promote the maintenance of active CAR T-cell proliferation following the initial infusion, ensuring continued effector cytotoxic anti-tumor activity.

In contrast, TCR-engineered T-cells differ structurally from CAR T-cells in that they use naturally occurring (or minimally modified) TCRs, lack co-stimulatory functions, and recognize peptide motifs bound to MHC [69] (Fig. 3).

One practical limitation is that TCR-transfected T-cell use is restricted to MHC proteins of certain HLA alleles—most frequently HLA-A*02:01—in clinical trials, as this is the most common HLA haplotype [70]. As a result, trial eligibility and future clinical utility will be restricted to patients whose HLA type has been “mapped” to a suitable MHC-presented antigen and for which there is a suitable TCR.

To date, CAR T-cells have demonstrated significant utility and are the basis of two approved therapeutics, tisagenleucel [71, 72], and axicabtagene ciloleucel [73, 74], which are limited to patients with hematologic malignancies expressing CD19 [75]. The first-generation CAR T-cell therapies capitalized on the unique restriction of CD19 expression to normal and malignant B-cells. There are very few lineage-specific surface protein markers similar to CD19 that can be used as targets in solid tumors. Because CARs harbor an extracellular antibody domain for T-cell antigen recognition, CAR T-cells can bind only to proteins expressed at the T-cell surface [73, 76, 77]. Lack of expression of antigens like CD19 intended for recognition by CAR T-cells has been associated with lack of response to these therapies [78].

Differences in toxicity profiles are also distinguishing features of CAR T-cells and TCR T-cells. Both have toxicities resulting from cancer-specific T-cell engagement (e.g., cytokine release syndrome). Alternatively, “on-target, off-tumor” toxicity occurs when the target antigens are expressed on non-malignant cells. This varies with the specificity of the CAR or TCR [79]. Depending on the specific CAR being employed, B-cell aplasia (generally limited to CARs binding CD19, CD20, and CD22) [80], cytokine release syndrome (on-target inflammation associated with IL-6-mediated T-cell activation) [81, 82], and central nervous system toxicity [82, 83] have been observed with CAR T-cells. Dermal, ocular, oto-, and cardiac toxicities are serious adverse outcomes that have been reported in patients receiving TCR T-cells [84, 85]. Toxicities associated with both CAR T-cell and TCR T-cell therapy can be serious and potentially life-threatening, and patients receiving these adoptive T-cell therapies require close observation by experienced providers in order to ensure prompt recognition and management of toxicities.

A major shortcoming of CAR-T cells is that they only target surface protein antigens, which are commonly not expressed on solid tumors. However, in addition to surface antigens, TCRs can target the commonly expressed intracellular antigens of solid tumors. Thus, TCRs offer an improved/expanded ability to address a wider variety of malignant diseases. Regarding the costs of these treatments, the TCR-transduced T-cell therapies are still investigational, and therefore, they are partially covered by the sponsors of the ongoing clinical trials. Therefore, their total cost cannot be compared to that of the FDA-approved CAR T-cell therapies. It is plausible that there would be substantially lower costs associated with the use of TCR-transduced T-cell therapies, as the manufacturing component is less arduous [86].

### Description and comparison of biotechnological approaches

ACT has expanded from a largely academic topic of investigation to becoming a focus of intense pharmaceutical company research and investment. Table 2 (as of August 2020) summarizes lines of investigation and products that are being developed by companies to treat solid tumors using TCR-based genetic modification of immune effector cells. Adaptimmune is currently developing four TCR-based adoptive therapy products primarily targeting MAGE and alpha-fetoprotein (AFP) peptide antigens expressed in solid cancers. The therapeutic indications include synovial carcinoma, hepatocellular carcinoma, non-small cell lung cancer (NSCLC), melanoma, and head and neck cancer. Bluebird Bio is advancing products that target the polyomavirus viral oncoprotein in patients with Merkel cell carcinoma; and in collaboration with Medigene is developing T-cell products that target MAGE-A4-expressing tumor cells. Immatics currently has three ACTengine® adoptive T-cell therapy programs in clinical development addressing patients with several solid tumor indications, including, but not limited to, head and neck squamous cell carcinoma, squamous NSCLC, hepatocellular carcinoma, uterine cancer, ovarian cancer, melanoma, and subtypes of sarcoma (Table 2): IMA201 targeting MAGE-A4 or MAGE-A8 antigen expressed in various solid tumors, IMA202 specific for MAGE-A1 in diverse solid cancers, and IMA203 targeting a PRAME antigen expressed in a broad range of solid tumors. Juno, a subsidiary of Bristol-Myers Squibb, is developing JTCR016, which targets WT1, focusing on NSCLC and mesothelioma. TCR^2^ therapeutics is developing TC-210, a mesothelin-targeted therapy for ovarian cancer, NSCLC, and cholangiocarcinoma. Tmunity has its H3.3K27M TCR program that focuses on patients with diffuse intrinsic pontine glioma. TScan therapeutics is developing the TCR TSC200 pipeline program targeting solid tumors. And, finally, Ziopharm is moving forward with its “Sleeping Beauty” TCR-T technology programs targeting NY-ESO-1 antigens in patients with multiple solid tumors. Tumor markers used for TCR-based therapy, their functions, and the tumor types associated with their overexpression are listed in Table 3 (as of August 2020). Collectively, the breadth of the approaches being taken will provide ample opportunity to elucidate the role of TCR-based therapies in anticancer therapy and focus on developing those with the greatest potential.

**Table 2:** Pipeline development: TCR-based therapy programs and their targets

Company/institution	TCR program(s) (investigational)	Target	Indication(s)	Key features
Adaptimmune	ADP-A2M4 SPEAR T-cells	MAGE-A4	Synovial Sarcoma	TCR (ADP-A2M4) targeting metastatic or inoperable (advanced) Synovial Sarcoma or MRCLS who have received prior chemotherapy and whose tumor expresses the MAGE-A4 tumor antigen. Evaluating urothelial (bladder) cancers, melanoma, head and neck cancer, ovarian cancer, NSCLC, esophageal cancer, gastric cancers, synovial sarcoma, and Myxoid Round Cell Liposarcoma (MRCLS). Adapted to mixed solid tumors secondary studies
Adaptimmune	ADP-A2M4CD8 SPEAR T-cell	MAGE-A4	Solid Tumors	TCR (ADP-A2M4CD8) which also expresses the CD8α co-receptor alongside the engineered TCR that targets MAGE-A4. Preclinical data indicate that co-expression of CD8α may broaden the immune response against solid tumors and increase antitumor activity by leveraging CD4 + cells into CD8 + killer or cytotoxic T-cells while retaining their CD4 + helper function
Adaptimmune	ADP-A2M10 T-cell	MAGE-A10	NSCLC, Melanoma, Bladder, Head and Neck	TCR (ADP-A2M10) targeting MAGE-A10 with potential ability to bind target peptides from multiple cancer types
Adaptimmune	ADP-A2AFP SPEAR T-cell	AFP	Hepatocellular Carcinoma	TCR (ADP-A2AFP) in SPEAR T-cell product which targets alpha-fetoprotein (AFP). Currently in Phase I clinical trial for the treatment of patients with hepatocellular carcinoma (liver cancer)
Bluebird Bio	MCC1 TCR	MCPyV	Merkel cell carcinoma	Autologous CD4 + and CD62L-expressing CD8 + T-cells expressing the high affinity T-cell receptor (TCR) A2-MCC1, specific for the human leucocyte antigen (HLA)-A02-restricted Merkel cell polyomavirus (MCPyV; MCV) viral oncoprotein. Final product is a cytotoxic T-lymphocyte (CTL) that targets tumor cells expressing the MCPyV viral oncoprotein. MCPyV viral oncoprotein is highly expressed in Merkel cell carcinoma (MCC) caused by MCPyV
Bluebird Bio/Medigene	MAGE-A4 TCR	MAGE-A4	Solid Tumors/Melanoma	Autologous human T lymphocytes transduced with MAGE-A4 as a co-receptor-independent TCR. After isolation, transduction, expansion, and reintroduction, MAGE-A4-specific TCR gene-transduced T lymphocytes bind to tumor cells expressing MAGE-A4. Effecting mechanism both inhibiting growth and increased cell death for MAGE-A4-expressing tumor cells. MAGE-A4 is overexpressed by a variety of cancer cell types
Kite/Gilead Sciences	KITE-718	MAGE-A3 and/or MAGE-A6	Solid Tumors/Advanced Cancers	Genetically modified T-cells which target tumor cells that express MAGE-A3 and/or MAGE-A6 in patients with solid tumors with relapsed or refractory disease after a systemic standard of care treatment
Kite/Gilead Sciences	KITE-439	HPV16 E6 and HPV16 E7	Solid Tumors/Advanced Cancers	Genetically modified T-cells which target cells that express HPV16 + solid tumors in patients with relapsed or refractory disease after at least 1 line of therapy that included systemic chemotherapy and not amenable to locoregional definitive therapy
Kite/Gilead Sciences	KITE-439	HPV16hat HOV	Solid Tumors/Advanced Cancers	Genetically modified T-cells which target cells that express HPV16 + solid tumors in patients with relapsed or refractory disease after at least 1 line of therapy that included systemic chemotherapy and not amenable to locoregional definitive therapy
Immatics	IMA201-101	MAGE-A4/8	Solid Tumors	ACTengine IMA201 genetically engineered T-cells (TCR-T) targeting MAGE-A4 or MAGE-A8 in patients with various solid tumors, including HNSCC, squamous NSCLC, subtypes of sarcoma and other solid tumor indications
Immatics	IMA202-101	MAGE-A1	Solid Tumors	ACTengine IMA202 genetically engineered T-cells (TCR-T) targeting MAGEA1 in patients with diverse solid tumors, including squamous NSCLC, hepatocellular carcinoma (HCC) and others
Immatics	IMA203-101	PRAME	Solid Tumors	ACTengine IMA203 genetically engineered T-cells (TCR-T) targeting PRAME in patients with a broad range of solid tumor types, including uterine cancer, ovarian cancer, melanoma, subtypes of sarcoma, squamous NSCLC and others
Juno	JTCR016	WT1	Stage III/IV NSCLC Mesothelioma	Autologous CD8 + T-cells genetically-modified to express a high affinity WT1-specific T-cell receptor targeting tumors in patients with stage III-IV non-small cell lung cancer (NSCLC) or mesothelioma
TCR^2^ Therapeutics	TC-210	Mesothelin	Ovarian Cancer, NSCLC, MPM, Cholangiocarcinoma	TCR-based adoptive therapy which targets mesothelin-positive solid tumors. Mesothelin is highly expressed in solid tumors and has a correlation with poor prognosis and tumorigenesis
Tmunity	NY-ESO-1 TCR-T Triple Knockout TCR (NYCE)	NY-ESO-1	Melanoma/Synovial Sarcoma	TCR-based adoptive therapy (NYCE) targeting NY-ESO-1 with designated target-binding capacity in melanoma and synovial sarcoma tumor types
Tmunity	H3.3K27M TCR	H3.3K27M	Diffuse intrinsic pontine glioma	Human T-cells transduced with a TCR that specifically targets the H3.3.K27M epitope and kills HLA-A2 + H3.3.K27M + glioma cells both in vitro and in vivo
Ziopharm	Sleeping Beauty TCR-T Targeting Neoantigens	NY-ESO-1 Personalized TCR-T (3 programs)	Multiple Solid Tumors	Genetically modified TCR therapies that target neoantigens. Sleeping Beauty’s non-viral (transposon/transposase) gene transfer system is suited for developing genetically modified TCR therapies that target neoantigens because of its very rapid manufacturing capability

**Table 3:** Selected tumor markers used for TCR-based therapy, function, and tumor types associated with their overexpression

Marker	Abbreviation	Function	Tumors associated with overexpression
AFP	Alpha Fetoprotein	Fetal development [109]—binds metals, fatty acids, and bilirubin	Hepatocellular carcinoma [110], testicular cancer [111]
H3.3K27M	Histone H3 trimethylation	Histone protein associated with aberrant chromatin compaction and silencing of tumor suppressor genes [112]	Prostate cancer [113], diffuse intrinsic pontine glioma [114]
HPV-16 E6	Human Papilloma Virus-16 E6	Oncoprotein that disrupts p53 function	Head/neck [115], cervix [116], anal canal [117]
HPV-16 E7	Human Papilloma Virus-16 E7	Oncoprotein that disrupts pRB function	Head/neck [115], cervix [116], anal canal [117]
MAGE-A1	Melanoma-associated antigen 1	Embryonic development, transcriptional regulation [118]	Non-small cell lung carcinoma [119]
MAGE-A3	Melanoma-associated antigen 3	Enhancement of E3 ubiquitin ligase activity [120]	Non-small cell lung carcinoma, melanoma [121], urothelial [122]
MAGE-A4	Melanoma-associated antigen 4	Embryonic development [123]	Non-small cell lung carcinoma [124], urothelial [125]
MAGE-A6	Melanoma-associated antigen 6	Enhancement of E3 ubiquitin ligase activity [126]	Breast [127], gastric [128]
MAGE-A8	Melanoma-associated antigen 8	Embryonic development [129]	Melanoma [130], urothelial [131]
MAGE-A10	Melanoma-associated antigen 10	Embryonic development [129]	Non-small cell lung carcinoma, melanoma, urothelial [132]
MCPyVs	Merkel cell polyoma virus (MCV oncoprotein)	Oncovirus integrates into infected cells	Merkel cell carcinoma [133]
Mesothelin	–	Cellular adhesion [134]	Mesothelioma [135], ovarian [136], pancreatic [137]
NY-ESO-1	Cancer/testis antigen 1	Embryonal development [138]	Melanoma [139], breast [140], ovarian [141], non-small cell lung carcinoma [142]
PRAME	Preferentially expressed antigen in melanoma	Transcriptional repressor	Melanoma [143], head/neck [144], osteosarcoma [145]
WT-1	Wilms tumor 1	Urogenital development [146]	Kidney [147], breast [148], leukemia [149]

### Clinical trials and patient outcomes

Adoptive T-cell therapy in selected studies is associated with high rates of durable complete response (CR) in patients with hematologic malignancies, even those with refractory disease [72, 73, 77]. Promising results have been reported with TILs in metastatic melanoma [33, 87–89], nasopharyngeal cancer [90], and cervical carcinoma [91]. The results of a comprehensive search of the National Institutes of Health (NIH) clinical trials database for engineered TCR-based therapies in solid tumors are presented in Table 4, and the key published clinical results from several companies and institutions are discussed in this section. Most published ACT trials use TCRs directed toward lineage-specific antigens, such as gp100 or Melan-A/MART-1, that may also be expressed by normal tissues at low levels. Alternatively, in other ACT trials a limited number of validated cancer germline antigens such as MAGE-A3 and NY-ESO-1, which are expressed in tumors, have been evaluated [92, 93].

**Table 4:** Selected TCR-based clinical trials for solid tumors

Sponsors/institutions	Indication	Treatment/target	Countries (# of sites)	NCT trial number
Adaptimmune	Solid tumors	MAGE-A4^c1032^T-cells	USA/Canada (9)	NCT03132922
Adaptimmune	Solid tumors	ADP-A2M4CD8 cells	USA/Belgium/Canada/Spain (16)	NCT04044859
Adaptimmune	Synovial sarcoma/myxoid liposarcoma	ADP-A2M4 cells	USA/France/Spain/UK (25)	NCT04044768
Adaptimmune	HCC	AFP^c332^T-cells	USA/France/Spain/UK (20)	NCT03132792
Adaptimmune	Solid tumors	MAGE A10^c796^T-cells	USA/Canada/Spain (11)	NCT02989064
Adaptimmune	Ovarian cancer	NYESO-1^c259^T-cells	USA (5)	NCT01567891
Adaptimmune	Melanoma	NY-ESO-1^c259^T-cells	USA (2)	NCT01350401
Adaptimmune	NSCLC	MAGE A10^c796^T-cells	USA/Canada/Spain/UK (19)	NCT02592577
Adaptimmune	Urothelial cancer, melanoma, head and neck cancer, urothelial carcinoma	MAGE A10^c796^T-cells	USA/Canada/Spain (11)	NCT02989064
Bellicum Pharmaceuticals	AML, myelodysplastic syndrome, uveal melanoma	BPX-701 (PRAME-TCR) infusion	USA (3)	NCT02743611
FHCRC	NSCLC, mesothelioma	WT1-TCRc4 gene-transduced CD8-positive Tcm/Tn Lymphocytes	USA (1)	NCT02408016
FHCRC	Merkel cell cancer	FH-MCVA2TCR T-cells (MCPyV-Specific TCRs)	USA (1)	NCT03747484
GlaxoSmithKline	Neoplasms	Anti-NY-ESO-1/LAGE-1a infusion	USA (25)	NCT03709706
GlaxoSmithKline	Synovial sarcoma	NY-ESO-1^c259^ transduced T-cell infusion	USA (8)	NCT01343043
GlaxoSmithKline	Solid tumors	GSK3377794 (NY-ESO-1 specific TCR engineered) infusion	USA/Canada/Spain/UK (15)	NCT03967223

Sponsors/institutions	Disease	Treatment/target	Countries (# of sites)	NCT trial number
GlaxoSmithKline	NSCLC	NY-ESO-1^c259^T-cells	USA (3)	NCT02588612
GlaxoSmithKline	Myxoid/round cell liposarcoma	NY-ESO-1^c259^T-cells	USA (6)	NCT02992743
Immatics	Solid tumors	MAGEA4/8T-cells (IMA201)	USA (3)	NCT03247309
Immatics	Solid tumors	MAGE-A1 T-cells (IMA202)	USA/Germany (6)	NCT03441100
Immatics	Solid tumors	PRAME T-cells (IMA203)	USA/Germany (6)	NCT03686124
Kite/Gilead Sciences	Solid tumors	KITE-718 (genetically modified MAGE-A3/A6 TCR transduced autologous T-cells) Infusion	USA (12)	NCT03139370
Kite/Gilead Sciences	HPV16 + cancers	E7 T-cell infusion (KITE-439)	USA (8)	NCT03912831
NCI/NIH CC	GI cancers	Anti-KRAS G12D mTCR PBL infusion	USA (1)	NCT03745326
NCI/NIH CC	GI cancers	Anti-KRAS G12V mTCR PBL infusion	USA (1)	NCT03190941
NCI/NIH CC	Breast, cervical, renal, melanoma, bladder cancer	Anti-MAGE-A3 infusion	USA (1)	NCT02153905
NCI/NIH CC	Cervical, renal, urothelial, melanoma, breast cancer	Anti-MAGE-A3-DP4 infusion	USA (1)	NCT02111850
NCI/NIH CC	Melanoma	Anti-MART-1 F5 infusion	USA (1)	NCT00706992
NCI/NIH CC	Melanoma or other cancers overexpressing p53	Anti-p53 infusion	USA (1)	NCT00393029
NCI/NIH CC	HPV + Cancers|Vulvar Neoplasms	HPV-16 E7 (E7 TCR) infusion	USA (1)	NCT02858310
NCI/NIH CC	HPV16 + Oropharyngeal Neoplasms	E7 TCR T-cells	USA (1)	NCT04015336; NCT04044950
NCI/NIH CC	HPV-Associated Cancers	Anti HPV E6 cells	USA (1)	NCT02280811
NCI/NIH CC	Melanoma	Anti-gp100:154–162 TCR TIL or PBL	USA (1)	NCT00509496
NCI/NIH CC	Metastatic Cancers	PG13-MAGE-A3 TCR9W11 (anti-MAGE-A3/12 TCR) PBL	USA (1)	NCT01273181
NCI/NIH CC	Melanoma	Anti-gp100:154 TCR PBL and anti-MART-1 F5 TCR PBL	USA (1)	NCT00923195
PACT Pharma, Inc	Solid Tumors	NeoTCR-P1 T-cells	USA (6)	NCT03970382
Shenzhen Second People's Hospital	Multiple Myeloma|Metastatic Solid Cancers	Anti-NY-ESO-1 infusion	USA (1)	NCT02457650
Sun Yat-sen University	NPC (HLA-A2; HLA-A11, HLA-A24)	EBV LMP2 antigen-specific TCR T-cell infusion	China (1)	NCT03925896
Sun Yat-sen University	Sarcoma	TAEST16001 (NY-ESO-1-specific TCR) cells	China (1)	NCT03462316
Xinqiao Hospital of Chongqing	Solid Tumors	HPV E6-specific TCR-T-cells	China (1)	NCT03578406
Zhujiang Hospital	Solid Tumors	TAEST16001 (NY-ESO-1-specific TCR) infusion	China (1)	NCT03159585
Guangzhou Institute of Respiratory Disease	NSCLC	NY-ESO-1-specific TCR-T-cells	China (1)	NCT03029273
Roswell Park Cancer Institute	Solid Tumors	NY-ESO-1 CD4-TCR CD34 + HSC on day 0; NY-ESO-1-specific CD8-positive T lymphocytes IV between days 7 and 21	USA (1)	NCT03691376
Albert Einstein College of Medicine	Solid Tumors	Anti-ESO (cancer/test antigen) mTCR-transduced cells	USA (1)	NCT02774291
TCR^2^ Therapeutics	Solid Tumors	TC-210 T-cells	USA (5)	NCT03907852

*FHCRC* Fred Hutchinson Cancer Research Center, *NCI/NIH CC* National Cancer Institute/National Institutes of Health Clinical Center, *AML* acute myeloid leukemia, *GI* gastrointestinal, *HCC* hepatocellular carcinoma, *NPC* nasopharyngeal carcinoma, *NSCLC* non-small cell lung cancer

Clinical proof of concept has already been demonstrated for TCR-engineered, autologous T-cell therapy in multiple myeloma, [94] melanoma [95–97], and other solid malignancies [98, 99]. Some investigators demonstrated that adoptive transfer of NY-ESO-1^c259^ T-cells in 42 patients with synovial sarcoma (NCT01343043) was associated with an objective response rate of 35.7% (15 patients; CR 1; PR 14) by RECIST [100]. Prolonged persistence and functionality of these adoptively transferred T-cells was associated with prolonged responses in some patients [101].

Encouraging results have been reported in patients with metastatic HPV16-positive cancers treated with autologous genetically engineered T-cells expressing a TCR directed against HPV16E6, demonstrating objective responses and a favorable adverse events profile [102] (NCT02280811). TCR^2^ therapeutics has used a unique TCR fusion construct (TRuC) platform without the need for HLA matching. This approach could make TCR therapies accessible to patients regardless of HLA type and is currently being tested in a phase I clinical trial in patients with advanced solid tumors (NCT03907852).

### Challenges and opportunities

The sequence of events necessary to provide TCR-based adoptive therapy to a specific patient are complex and require a structured and integrated process. This process includes the screening of patients (for HLA typing and identification of the targeted tumor antigen); the evaluation of patient suitability for lymphodepletion; the isolation by leukapheresis of effector cells (e.g., lymphocytes); and the generation, expansion, infusion of the TCR-based adoptive therapy. Optimization of technology and treatment administration is required at every step of the process for successful TCR-based adoptive T-cell therapy (Fig. 1). Pharmacological and pharmacodynamic aspects of lymphodepletion should be considered. This sequence of events can take several weeks, making it inaccessible for many patients needing immediate therapy. In some cases, a bridging therapy can be used until the TCR therapeutic is available. The infusion and monitoring of patients for this therapy also has considerable complexity, as it may require the coordinated application of a lymphodepletion regimen, the TCR product, IL-2, supportive care, and close monitoring for cytokine release syndrome, which itself requires specific interventions (Fig. 1). The future of these personalized therapies requires making the products more efficient and generally applicable in routine patient care (Table 5).

**Table 5:** Challenges, opportunities, and future directions

Challenges	Current status	Opportunities/resolution
HLA Subtype Compatibility (HLA-A*02:01)	Therapies inclusive only to HLA-A*02:01 positive patients. Serotype is highly prevalent in Caucasian and native American populations yet low in other races and ethnicities	Broadening these therapies to multiple HLA genotypes and subtypes will increase the inclusivity and availability to a wider range of patients
Histological Biomarker Analyses	Costly and invasive tumor biopsy step needed to screen tumor tissue for confirmed expression of the targeted antigen	Develop new techniques to transcend current biopsy logistics and costs. Consider emerging circulating tumor cell techniques to identify target antigens
Identification and Selection of Target Antigens	Translational retroactive studies focusing on correlating data to identify suitable tumor antigens that are unique to a specific cancer and activate the immune response	Utilize bioinformatics technologies to develop predictive algorithms to identify effective target patient populations and tractable tumor antigens that enhance on-target, on-tumor immunocompetent responses and attenuate on-target off-tumor untoward effects
Leukapheresis Techniques and Manufacturing Starting Material	Current process is to extract and isolate PBMCs via standard apheresis techniques and utilized as the initial material for genetic modification	Advance apheresis techniques and improve autologous procedure technologies by enriching and activating T-cell subpopulations as the starting material
Temporal window from leukapheresis to product delivery	Current median times from leukapheresis to product delivery is 2–3 weeks	Augment and enhance the manufacturing, development, and delivery logistics processes to reduce the autologous extraction-to-infusion time frame
Pre-Infusion Lymphodepletion	Standard conditioning method supporting enhancement of engraftment and persistence of modified transferred T-cells	Fine tune and adapt the use of lymphodepletion agents to maximize immunocompetence and clinical benefit
Centralized Manufacturing/Processing Center	Present manufacturing methodology centralizes the preparation of TCR-based adoptive therapy at a core center to be subsequently returned and administered to the patient	Project to create regional or hospital-based centers where the extraction, modification, and infusion of the T-cell product occurs at the same location
Protracted Patient Follow-Up	Current regulatory guidance recommends patient follow-up for 15 years to screen for untoward long-term effects	Innovate post-administration safety assessments to efficiently monitor patients as well pioneering pre-infusion translational research studies that demonstrate the safety longevity of genetically-modified cells
Screening for optimal TCR affinity	Naturally occurring, tumor‐reactive T-cells might have poor efficacy because of the expression of low‐affinity TCRs	High affinity T-cells specific for candidate tumor antigens that are non-mutated self-antigens are likely candidates for such negative selection. Various strategies have been developed to enhance the affinity and the functional avidity of TCRs targeting tumor antigens. However, affinity‐enhanced TCRs might increase the risk of autoimmunity [150, 151]
Combination with checkpoint blockade	Immune checkpoint inhibitors, such as PD-1/PD-L1 and CTLA-4 along with other treatment modalities have been widely considered in the engineered TCR clinical trials	Approaches interfere with these inhibitory receptors are being tested to further enhance the antitumor activity of engineered T-cells [152–155]. Checkpoint inhibition could, if administered before T-cell harvest, may facilitate the T-cells to be used for ACT product manufacture. This type of treatment could potentially be used to improve the quality of ex vivo expanded T-cell immunotherapy [156]. However, increasing upregulated expression of inhibitory receptors may limit the anti-tumor response by T-cell exhaustion
TCR-edited T-cells	The CRISPR-engineered T-cells may facilitate recognition of tumor cells by deleting the endogenous TCRs and PD-1 to reduce T-cell exhaustion	CRISPR-Cas9 technology was used in an example as a synthetic, cancer specific TCR transgene (NY-ESO-1) to facilitate recognition of tumor cells by the engineered T-cells. T-cells expressing NY-ESO-1 and lacking PD-1 and endogenous TCR have sustained in vivo expansion and persistence in a pilot phase I trial, suggesting additional tumor antigens may be required to see full tumor response [157]

An ongoing limitation of many of the current studies is the need to restrict enrollment to HLA-A*02:01-positive patients. This HLA haplotype is prevalent in Caucasian (~ 40%) and Native American populations, yet not as common in other populations. Broadening these therapies to multiple HLA genotypes and subtypes will increase availability to a wider range of patients. To achieve this, new TCRs are currently being developed for a broad range of HLA haplotypes by several investigators. More importantly, TCR therapy is directed against specific tumor markers, with variable prevalence in selected tumor types. Discovery of novel tumor markers that are expressed in more patients and tumor types is needed to offer this strategy to more patients with solid tumors. Unfortunately, even with targetable antigens/markers, there are secondary lines of defense for solid tumors, such as altered cellular penetration and challenges related to the persistence of TCRs and to the tumor microenvironment itself, all of which need to be addressed for this treatment to become widely applicable going forward. The role of targeting the tumor microenvironment in addition to the malignant cells for tumor control has been previously highlighted [103, 104].

Currently, lymphodepletion is accomplished with chemotherapy (e.g., FC), as research findings support that lymphodepletion enhances treatment efficacy (by providing a favorable immune environment). Yet, lymphodepleting conditioning needs further optimization to make it safer and more broadly applicable.

Overall, genetically modified cell therapies are more arduous to administer and are associated with significant long-term risks. Consequently, the FDA has implemented stringent rules in clinical trials of genetically modified cell therapies (i.e., 15-year follow-up for monitoring the effects of genetic modifications). Due to the personalized nature of developing TCR therapy, several inherent technical challenges are associated with the quality and procurement of lymphocytes (from leukapheresis) and with the manufacturing and processing of the final TCR product. Advances in technology and standardization of lymphocyte manufacturing may increase the success rate of TCR therapy. The implementation of TCR therapy will require a shortened time to manufacture TCR products and decreased overall cost associated with the administration of TCR therapy. Additionally, since centralized production of T-cell products is expensive, it is plausible that smaller production facilities could be generated on a franchise-like basis where vectors and cell culture materials are supplied to the local T-cell production sites. In this direction, “bioreactors” (i.e., smaller contained production units) are being investigated in clinical trials [105, 106]. Theoretically, this expansion of TCR therapies may increase the success rate, yet it will require the training of highly specialized personnel, the establishment of Good Manufacturing Practices-certified facilities, and conformation to the same stringent FDA regulations that surround the production of TCR products.

It is essential to conquer the obstacles associated with the manufacturing and administration of TCR therapy, including those challenges posed by the immunosuppressive microenvironment in solid tumors, as well as to develop next-generation strategies designed to improve the efficacy and safety of TCR therapies [107]. Although current TCR therapies have the potential to cure selected patients who meet the criteria to receive these treatments, given that MHC-I is downregulated/deficient in 40–90% of patients, these treatments may not be suitable or efficacious for the majority of patients with solid tumors. TCRs are promising because there are more cancer antigens available inside the cells than on the surface, e.g., CAR-T cells can only target surface antigens, whereas engineered TCR-T cells will recognize and attack intracellular tumor-related antigens. These two approaches complement each other. Ongoing and future clinical trials will determine the role of TCR therapy in the armamentarium of therapeutic strategies against cancer.

## Conclusion

TCR-based adoptive cell therapies are currently being tested in a variety of advanced cancers with the results to date indicating that the technology is presumptively safe and prospectively efficacious. Such therapies will likely complement, not replace CAR-T-based therapies as their distinct attributes will further address unique aspects associated with the diverse solid tumor landscape. Many challenges need to be addressed to fully exploit TCR-based therapies, including those associated with TCR product manufacturing, patient selection, patient preparation with lymphodepletion, administration of treatment and monitoring of adverse events. Overcoming these challenges, and those posed by the immunosuppressive tumor microenvironment, as well as developing next-generation strategies are essential for improving the efficacy, safety and widespread applicability of TCR-based therapies. Ongoing and future clinical trials will determine the role of TCR therapy in patients with solid tumors.

## Data Availability

Not applicable.

## Abbreviations

ACT: Adoptive cell therapy
AFP: Alpha-fetoprotein
AML: Acute myeloid leukemia
APCs: Professional antigen-presenting cells
CAR: Chimeric antigen receptor
CLIP: Class II-associated Ii peptide
CR: Complete response
CTL: Cytotoxic T‑lymphocyte
FC: Fludarabine
FDA: Food and Drug Administration
FHCRC: Fred Hutchinson Cancer Research Center
GI: Gastrointestinal
HCC: Hepatocellular carcinoma
HLA: Human leukocyte antigen
IFN‐γ: Interferon-γ
IL-2: Interleukin 2
LD: Lymphodepletion
LV: Lentiviral
MAGE: Melanoma antigen gene
MCC: Merkel cell carcinoma
mFC: Modified FC
MHC: Major histocompatibility complex
MIIC: MHC class II compartment
NCI/NIH CC: National Cancer Institute/National Institutes of Health Clinical Center
NIH: National Institutes of Health
NK: Natural killer
NPC: Nasopharyngeal carcinoma
NSCLC: Non-small cell lung cancer
NY-ESO: New York esophageal squamous cell carcinoma
PBMC: Peripheral blood mononuclear cell
RV: Retroviral
TBI: Total body irradiation
TCR: T-cell receptor
TIL: Tumor-infiltrating lymphocyte
TNF: Tumor necrosis factor
XRT: Radiation therapy

## References

[1] ACTolog in Patients with Solid Cancers (ACTolog). https://clinicaltrials.gov/ct2/show/NCT02876510. Accessed Aug 2020.

[2] Ping Y, Liu C, Zhang Y (2018). T-cell receptor-engineered T cells for cancer treatment: current status and future directions. Protein Cell.

[3] Huang D, Miller M, Ashok B, Jain S, Peppas NA (2020). CRISPR/Cas systems to overcome challenges in developing the next generation of T cells for cancer therapy. Adv Drug Deliv Rev.

[4] Birnbaum ME, Berry R, Hsiao YS, Chen Z, Shingu-Vazquez MA, Yu X (2014). Molecular architecture of the alphabeta T cell receptor-CD3 complex. Proc Natl Acad Sci USA.

[5] Hewitt EW (2003). The MHC class I antigen presentation pathway: strategies for viral immune evasion. Immunology.

[6] Robbins PF, Li YF, El-Gamil M, Zhao Y, Wargo JA, Zheng Z (2008). Single and dual amino acid substitutions in TCR CDRs can enhance antigen-specific T cell functions. J Immunol.

[7] Atanackovic D, Altorki NK, Cao Y, Ritter E, Ferrara CA, Ritter G (2008). Booster vaccination of cancer patients with MAGE-A3 protein reveals long-term immunological memory or tolerance depending on priming. Proc Natl Acad Sci USA.

[8] van Baren N, Bonnet MC, Dreno B, Khammari A, Dorval T, Piperno-Neumann S (2005). Tumoral and immunologic response after vaccination of melanoma patients with an ALVAC virus encoding MAGE antigens recognized by T cells. J Clin Oncol.

[9] Green EW, Bunse L, Bozza M, Sanghvi K, Platten M (2019). TCR validation toward gene therapy for cancer. Methods Enzymol.

[10] Karapetyan AR, Chaipan C, Winkelbach K, Wimberger S, Jeong JS, Joshi B (2019). TCR fingerprinting and Off-target peptide identification. Front Immunol.

[11] Bentzen AK, Such L, Jensen KK, Marquard AM, Jessen LE, Miller NJ (2018). T cell receptor fingerprinting enables in-depth characterization of the interactions governing recognition of peptide-MHC complexes. Nat Biotechnol.

[12] Neefjes J, Jongsma ML, Paul P, Bakke O (2011). Towards a systems understanding of MHC class I and MHC class II antigen presentation. Nat Rev Immunol.

[13] Wooldridge L, van den Berg HA, Glick M, Gostick E, Laugel B, Hutchinson SL (2005). Interaction between the CD8 coreceptor and major histocompatibility complex class I stabilizes T cell receptor-antigen complexes at the cell surface. J Biol Chem.

[14] Laugel B, van den Berg HA, Gostick E, Cole DK, Wooldridge L, Boulter J (2007). Different T cell receptor affinity thresholds and CD8 coreceptor dependence govern cytotoxic T lymphocyte activation and tetramer binding properties. J Biol Chem.

[15] Hoffmann MM, Slansky JE (2020). T-cell receptor affinity in the age of cancer immunotherapy. Mol Carcinog.

[16] Huang J, Brameshuber M, Zeng X, Xie J, Li QJ, Chien YH (2013). A single peptide-major histocompatibility complex ligand triggers digital cytokine secretion in CD4(+) T cells. Immunity.

[17] van der Merwe PA, Dushek O (2011). Mechanisms for T cell receptor triggering. Nat Rev Immunol.

[18] Fulton RB, Hamilton SE, Xing Y, Best JA, Goldrath AW, Hogquist KA (2015). The TCR's sensitivity to self peptide-MHC dictates the ability of naive CD8(+) T cells to respond to foreign antigens. Nat Immunol.

[19] Rosenberg SA, Restifo NP (2015). Adoptive cell transfer as personalized immunotherapy for human cancer. Science.

[20] Ellis GI, Sheppard NC, Riley JL (2021). Genetic engineering of T cells for immunotherapy. Nat Rev Genet.

[21] Chandran SS, Klebanoff CA (2019). T cell receptor-based cancer immunotherapy: Emerging efficacy and pathways of resistance. Immunol Rev.

[22] Bubenik J (2003). Tumour MHC class I downregulation and immunotherapy (review). Oncol Rep.

[23] Garrido F, Aptsiauri N, Doorduijn EM, Garcia Lora AM, van Hall T (2016). The urgent need to recover MHC class I in cancers for effective immunotherapy. Curr Opin Immunol.

[24] Gilboa E (1999). The makings of a tumor rejection antigen. Immunity.

[25] Schumacher TN, Schreiber RD (2015). Neoantigens in cancer immunotherapy. Science.

[26] Ward JP, Gubin MM, Schreiber RD (2016). The role of neoantigens in naturally occurring and therapeutically induced immune responses to cancer. Adv Immunol.

[27] Yarchoan M, Johnson BA, Lutz ER, Laheru DA, Jaffee EM (2017). Targeting neoantigens to augment antitumour immunity. Nat Rev Cancer.

[28] Jiang T, Shi T, Zhang H, Hu J, Song Y, Wei J (2019). Tumor neoantigens: from basic research to clinical applications. J Hematol Oncol.

[29] Klebanoff CA, Khong HT, Antony PA, Palmer DC, Restifo NP (2005). Sinks, suppressors and antigen presenters: how lymphodepletion enhances T cell-mediated tumor immunotherapy. Trends Immunol.

[30] Dudley ME, Yang JC, Sherry R, Hughes MS, Royal R, Kammula U (2008). Adoptive cell therapy for patients with metastatic melanoma: evaluation of intensive myeloablative chemoradiation preparative regimens. J Clin Oncol.

[31] Uttenthal BJ, Chua I, Morris EC, Stauss HJ (2012). Challenges in T cell receptor gene therapy. J Gene Med.

[32] Muranski P, Boni A, Wrzesinski C, Citrin DE, Rosenberg SA, Childs R (2006). Increased intensity lymphodepletion and adoptive immunotherapy–how far can we go?. Nat Clin Pract Oncol.

[33] Besser MJ, Shapira-Frommer R, Itzhaki O, Treves AJ, Zippel DB, Levy D (2013). Adoptive transfer of tumor-infiltrating lymphocytes in patients with metastatic melanoma: intent-to-treat analysis and efficacy after failure to prior immunotherapies. Clin Cancer Res.

[34] Dudley ME, Wunderlich JR, Yang JC, Sherry RM, Topalian SL, Restifo NP (2005). Adoptive cell transfer therapy following non-myeloablative but lymphodepleting chemotherapy for the treatment of patients with refractory metastatic melanoma. J Clin Oncol.

[35] Yamauchi T, Nowak BJ, Keating MJ, Plunkett W (2001). DNA repair initiated in chronic lymphocytic leukemia lymphocytes by 4-hydroperoxycyclophosphamide is inhibited by fludarabine and clofarabine. Clin Cancer Res.

[36] Valdez BC, Andersson BS (2010). Interstrand crosslink inducing agents in pretransplant conditioning therapy for hematologic malignancies. Environ Mol Mutagen.

[37] Long-Boyle JR, Green KG, Brunstein CG, Cao Q, Rogosheske J, Weisdorf DJ (2011). High fludarabine exposure and relationship with treatment-related mortality after nonmyeloablative hematopoietic cell transplantation. Bone Marrow Transplant.

[38] Sanghavi K, Wiseman A, Kirstein MN, Cao Q, Brundage R, Jensen K (2016). Personalized fludarabine dosing to reduce nonrelapse mortality in hematopoietic stem-cell transplant recipients receiving reduced intensity conditioning. Transl Res.

[39] Rudolph MG, Stanfield RL, Wilson IA (2006). How TCRs bind MHCs, peptides, and coreceptors. Annu Rev Immunol.

[40] Hovav AH (2017). Human gammadelta T cells: rapid, stable and clonally reactive. Cell Mol Immunol.

[41] Fisher JP, Yan M, Heuijerjans J, Carter L, Abolhassani A, Frosch J (2014). Neuroblastoma killing properties of Vdelta2 and Vdelta2-negative gammadeltaT cells following expansion by artificial antigen-presenting cells. Clin Cancer Res.

[42] Sebestyen Z, Prinz I, Dechanet-Merville J, Silva-Santos B, Kuball J (2020). Translating gammadelta (gammadelta) T cells and their receptors into cancer cell therapies. Nat Rev Drug Discov.

[43] Chien YH, Meyer C, Bonneville M (2014). gammadelta T cells: first line of defense and beyond. Annu Rev Immunol.

[44] Ma Y, Aymeric L, Locher C, Mattarollo SR, Delahaye NF, Pereira P (2011). Contribution of IL-17-producing gamma delta T cells to the efficacy of anticancer chemotherapy. J Exp Med.

[45] Fisher J, Anderson J (2018). Engineering approaches in human gamma delta T cells for cancer immunotherapy. Front Immunol.

[46] Maude SL, Frey N, Shaw PA, Aplenc R, Barrett DM, Bunin NJ (2014). Chimeric antigen receptor T cells for sustained remissions in leukemia. N Engl J Med.

[47] Sadelain M, Riviere I, Riddell S (2017). Therapeutic T cell engineering. Nature.

[48] Berdien B, Mock U, Atanackovic D, Fehse B (2014). TALEN-mediated editing of endogenous T-cell receptors facilitates efficient reprogramming of T lymphocytes by lentiviral gene transfer. Gene Ther.

[49] Bendle GM, Linnemann C, Hooijkaas AI, Bies L, de Witte MA, Jorritsma A (2010). Lethal graft-versus-host disease in mouse models of T cell receptor gene therapy. Nat Med.

[50] Cohen CJ, Zhao Y, Zheng Z, Rosenberg SA, Morgan RA (2006). Enhanced antitumor activity of murine-human hybrid T-cell receptor (TCR) in human lymphocytes is associated with improved pairing and TCR/CD3 stability. Cancer Res.

[51] Dorrie J, Krug C, Hofmann C, Muller I, Wellner V, Knippertz I (2014). Human adenovirus-specific gamma/delta and CD8+ T cells generated by T-cell receptor transfection to treat adenovirus infection after allogeneic stem cell transplantation. PLoS ONE.

[52] Parlar A, Sayitoglu EC, Ozkazanc D, Georgoudaki AM, Pamukcu C, Aras M (2019). Engineering antigen-specific NK cell lines against the melanoma-associated antigen tyrosinase via TCR gene transfer. Eur J Immunol.

[53] Kim S, Poursine-Laurent J, Truscott SM, Lybarger L, Song YJ, Yang L (2005). Licensing of natural killer cells by host major histocompatibility complex class I molecules. Nature.

[54] Davies JOJ, Stringaris K, Barrett AJ, Rezvani K (2014). Opportunities and limitations of natural killer cells as adoptive therapy for malignant disease. Cytotherapy.

[55] Wagner JA, Berrien-Elliott MM, Rosario M, Leong JW, Jewell BA, Schappe T (2017). Cytokine-induced memory-like differentiation enhances unlicensed natural killer cell Antileukemia and FcgammaRIIIa-triggered responses. Biol Blood Marrow Transplant.

[56] Rezvani K, Rouce R, Liu E, Shpall E (2017). Engineering natural killer cells for cancer immunotherapy. Mol Ther.

[57] Dahlberg CI, Sarhan D, Chrobok M, Duru AD, Alici E (2015). Natural killer cell-based therapies targeting cancer: possible strategies to gain and sustain anti-tumor activity. Front Immunol.

[58] Pegram HJ, Kershaw MH, Darcy PK (2009). Genetic modification of natural killer cells for adoptive cellular immunotherapy. Immunotherapy.

[59] Uherek C, Tonn T, Uherek B, Becker S, Schnierle B, Klingemann HG (2002). Retargeting of natural killer-cell cytolytic activity to ErbB2-expressing cancer cells results in efficient and selective tumor cell destruction. Blood.

[60] Atkins MB, Kunkel L, Sznol M, Rosenberg SA (2000). High-dose recombinant interleukin-2 therapy in patients with metastatic melanoma: long-term survival update. Cancer J Sci Am.

[61] Rosenberg SA (2014). IL-2: the first effective immunotherapy for human cancer. J Immunol.

[62] Rosenberg SA, Yannelli JR, Yang JC, Topalian SL, Schwartzentruber DJ, Weber JS (1994). Treatment of patients with metastatic melanoma with autologous tumor-infiltrating lymphocytes and interleukin 2. J Natl Cancer Inst.

[63] Yee C, Thompson JA, Byrd D, Riddell SR, Roche P, Celis E (2002). Adoptive T cell therapy using antigen-specific CD8+ T cell clones for the treatment of patients with metastatic melanoma: in vivo persistence, migration, and antitumor effect of transferred T cells. Proc Natl Acad Sci USA.

[64] Chapuis AG, Ragnarsson GB, Nguyen HN, Chaney CN, Pufnock JS, Schmitt TM (2013). Transferred WT1-reactive CD8+ T cells can mediate antileukemic activity and persist in post-transplant patients. Sci Transl Med.

[65] Kuwana Y, Asakura Y, Utsunomiya N, Nakanishi M, Arata Y, Itoh S (1987). Expression of chimeric receptor composed of immunoglobulin-derived V regions and T-cell receptor-derived C regions. Biochem Biophys Res Commun.

[66] Eshhar Z, Waks T, Gross G, Schindler DG (1993). Specific activation and targeting of cytotoxic lymphocytes through chimeric single chains consisting of antibody-binding domains and the gamma or zeta subunits of the immunoglobulin and T-cell receptors. Proc Natl Acad Sci USA.

[67] Bridgeman JS, Hawkins RE, Bagley S, Blaylock M, Holland M, Gilham DE (2010). The optimal antigen response of chimeric antigen receptors harboring the CD3zeta transmembrane domain is dependent upon incorporation of the receptor into the endogenous TCR/CD3 complex. J Immunol.

[68] Guedan S, Posey AD, Shaw C, Wing A, Da T, Patel PR (2018). Enhancing CAR T cell persistence through ICOS and 4-1BB costimulation. JCI Insight.

[69] Sun ZJ, Kim KS, Wagner G, Reinherz EL (2001). Mechanisms contributing to T cell receptor signaling and assembly revealed by the solution structure of an ectodomain fragment of the CD3 epsilon gamma heterodimer. Cell.

[70] Song S, Han M, Zhang H, Wang Y, Jiang H (2013). Full screening and accurate subtyping of HLA-A*02 alleles through group-specific amplification and mono-allelic sequencing. Cell Mol Immunol.

[71] Schuster SJ, Bishop MR, Tam CS, Waller EK, Borchmann P, McGuirk JP (2019). Tisagenlecleucel in adult relapsed or refractory diffuse large B-cell lymphoma. N Engl J Med.

[72] Maude SL, Laetsch TW, Buechner J, Rives S, Boyer M, Bittencourt H (2018). Tisagenlecleucel in children and young adults with B-cell lymphoblastic leukemia. N Engl J Med.

[73] Neelapu SS, Locke FL, Bartlett NL, Lekakis LJ, Miklos DB, Jacobson CA (2017). Axicabtagene ciloleucel CAR T-cell therapy in refractory large B-cell lymphoma. N Engl J Med.

[74] Locke FL, Ghobadi A, Jacobson CA, Miklos DB, Lekakis LJ, Oluwole OO (2019). Long-term safety and activity of axicabtagene ciloleucel in refractory large B-cell lymphoma (ZUMA-1): a single-arm, multicentre, phase 1–2 trial. Lancet Oncol.

[75] June CH, Sadelain M (2018). Chimeric antigen receptor therapy. N Engl J Med.

[76] Schuster SJ, Svoboda J, Chong EA, Nasta SD, Mato AR, Anak O (2017). Chimeric antigen receptor T cells in refractory B-cell lymphomas. N Engl J Med.

[77] Wang M, Munoz J, Goy A, Locke FL, Jacobson CA, Hill BT (2020). KTE-X19 CAR T-cell therapy in relapsed or refractory mantle-cell lymphoma. N Engl J Med.

[78] Sotillo E, Barrett DM, Black KL, Bagashev A, Oldridge D, Wu G (2015). Convergence of acquired mutations and alternative splicing of CD19 enables resistance to CART-19 immunotherapy. Cancer Discov.

[79] Harris DT, Kranz DM (2016). Adoptive T cell therapies: a comparison of T cell receptors and chimeric antigen receptors. Trends Pharmacol Sci.

[80] LeBien TW, Tedder TF (2008). B lymphocytes: how they develop and function. Blood.

[81] Paszkiewicz PJ, Frassle SP, Srivastava S, Sommermeyer D, Hudecek M, Drexler I (2016). Targeted antibody-mediated depletion of murine CD19 CAR T cells permanently reverses B cell aplasia. J Clin Investig.

[82] Brudno JN, Kochenderfer JN (2019). Recent advances in CAR T-cell toxicity: mechanisms, manifestations and management. Blood Rev.

[83] Lee DW, Santomasso BD, Locke FL, Ghobadi A, Turtle CJ, Brudno JN (2019). ASTCT consensus grading for cytokine release syndrome and neurologic toxicity associated with immune effector cells. Biol Blood Marrow Transplant.

[84] Overwijk WW, Theoret MR, Finkelstein SE, Surman DR, de Jong LA, Vyth-Dreese FA (2003). Tumor regression and autoimmunity after reversal of a functionally tolerant state of self-reactive CD8+ T cells. J Exp Med.

[85] Palmer DC, Chan CC, Gattinoni L, Wrzesinski C, Paulos CM, Hinrichs CS (2008). Effective tumor treatment targeting a melanoma/melanocyte-associated antigen triggers severe ocular autoimmunity. Proc Natl Acad Sci USA.

[86] Lyman GH, Nguyen A, Snyder S, Gitlin M, Chung KC (2020). Economic evaluation of chimeric antigen receptor T-cell therapy by site of care among patients with relapsed or refractory large B-cell lymphoma. JAMA Netw Open.

[87] Rosenberg SA, Yang JC, Sherry RM, Kammula US, Hughes MS, Phan GQ (2011). Durable complete responses in heavily pretreated patients with metastatic melanoma using T-cell transfer immunotherapy. Clin Cancer Res.

[88] Andersen R, Donia M, Ellebaek E, Borch TH, Kongsted P, Iversen TZ (2016). Long-lasting complete responses in patients with metastatic melanoma after adoptive cell therapy with tumor-infiltrating lymphocytes and an attenuated IL2 regimen. Clin Cancer Res.

[89] Forget MA, Haymaker C, Hess KR, Meng YJ, Creasy C, Karpinets T (2018). Prospective analysis of adoptive TIL therapy in patients with metastatic melanoma: response, impact of anti-CTLA4, and biomarkers to predict clinical outcome. Clin Cancer Res.

[90] Comoli P, Pedrazzoli P, Maccario R, Basso S, Carminati O, Labirio M (2005). Cell therapy of stage IV nasopharyngeal carcinoma with autologous Epstein-Barr virus-targeted cytotoxic T lymphocytes. J Clin Oncol.

[91] Stevanovic S, Draper LM, Langhan MM, Campbell TE, Kwong ML, Wunderlich JR (2015). Complete regression of metastatic cervical cancer after treatment with human papillomavirus-targeted tumor-infiltrating T cells. J Clin Oncol.

[92] Cheever MA, Allison JP, Ferris AS, Finn OJ, Hastings BM, Hecht TT (2009). The prioritization of cancer antigens: a national cancer institute pilot project for the acceleration of translational research. Clin Cancer Res.

[93] Debets R, Donnadieu E, Chouaib S, Coukos G (2016). TCR-engineered T cells to treat tumors: seeing but not touching?. Semin Immunol.

[94] Rapoport AP, Stadtmauer EA, Binder-Scholl GK, Goloubeva O, Vogl DT, Lacey SF (2015). NY-ESO-1-specific TCR-engineered T cells mediate sustained antigen-specific antitumor effects in myeloma. Nat Med.

[95] Johnson LA, Morgan RA, Dudley ME, Cassard L, Yang JC, Hughes MS (2009). Gene therapy with human and mouse T-cell receptors mediates cancer regression and targets normal tissues expressing cognate antigen. Blood.

[96] Morgan RA, Dudley ME, Wunderlich JR, Hughes MS, Yang JC, Sherry RM (2006). Cancer regression in patients after transfer of genetically engineered lymphocytes. Science.

[97] Robbins PF, Kassim SH, Tran TL, Crystal JS, Morgan RA, Feldman SA (2015). A pilot trial using lymphocytes genetically engineered with an NY-ESO-1-reactive T-cell receptor: long-term follow-up and correlates with response. Clin Cancer Res.

[98] Parkhurst MR, Yang JC, Langan RC, Dudley ME, Nathan DA, Feldman SA (2011). T cells targeting carcinoembryonic antigen can mediate regression of metastatic colorectal cancer but induce severe transient colitis. Mol Ther.

[99] Lu Y-C, Parker L, Lu T, Zheng Z, Yao X, Robbins PF (2015). A Phase I study of an HLA-DPB1*0401-restricted T cell receptor targeting MAGE-A3 for patients with metastatic cancers. J ImmunoTherapy Cancer.

[100] Ramachandran I, Lowther DE, Dryer-Minnerly R, Wang R, Fayngerts S, Nunez D (2019). Systemic and local immunity following adoptive transfer of NY-ESO-1 SPEAR T cells in synovial sarcoma. J Immunother Cancer.

[101] D'Angelo SP, Melchiori L, Merchant MS, Bernstein D, Glod J, Kaplan R (2018). Antitumor activity associated with prolonged persistence of adoptively transferred NY-ESO-1 (c259)T cells in synovial sarcoma. Cancer Discov.

[102] Doran SL, Stevanovic S, Adhikary S, Gartner JJ, Jia L, Kwong MLM (2019). T-cell receptor gene therapy for human papillomavirus-associated epithelial cancers: a first-in-human, Phase I/II Study. J Clin Oncol.

[103] Ruella M, Klichinsky M, Kenderian SS, Shestova O, Ziober A, Kraft DO (2017). Overcoming the immunosuppressive tumor microenvironment of Hodgkin lymphoma using chimeric antigen receptor T cells. Cancer Discov.

[104] Rajasekaran AK, Zhou Z, Prakash K, Das G, Kreibich G (1995). Functional characterization of the cis-regulatory elements of the rat ribophorin I gene. Nucleic Acids Res.

[105] Gagliardi C, Khalil M, Foster AE (2019). Streamlined production of genetically modified T cells with activation, transduction and expansion in closed-system G-Rex bioreactors. Cytotherapy.

[106] Langford S, Bowersock J, Pillai S, Lamb LS (2017). Manufacturing of expanded/activated γδ T cells using the Miltenyi Prodigy® bioreactor system. Cytotherapy.

[107] Morotti M, Albukhari A, Alsaadi A, Artibani M, Brenton JD, Curbishley SM (2021). Promises and challenges of adoptive T-cell therapies for solid tumours. Br J Cancer.

[108] Liu SQ, Grantham A, Landry C, Granda B, Chopra R, Chakravarthy S (2021). A CRISPR screen reveals resistance mechanisms to CD3-bispecific antibody therapy. Cancer Immunol Res.

[109] Crandall BF (1981). Alpha-fetoprotein: a review. Crit Rev Clin Lab Sci.

[110] Masopust J, Kithier K, Radl J, Koutecky J, Kotal L (1968). Occurrence of fetoprotein in patients with neoplasms and non-neoplastic diseases. Int J Cancer.

[111] Germa-Lluch JR, GarciadelMuro X, Maroto P, Paz-Ares L, Arranz JA, Guma J (2002). Clinical pattern and therapeutic results achieved in 1490 patients with germ-cell tumours of the testis: the experience of the Spanish Germ-Cell Cancer Group (GG). Eur Urol.

[112] Margueron R, Reinberg D (2011). The Polycomb complex PRC2 and its mark in life. Nature.

[113] Ngollo M, Lebert A, Dagdemir A, Judes G, Karsli-Ceppioglu S, Daures M (2014). The association between histone 3 lysine 27 trimethylation (H3K27me3) and prostate cancer: relationship with clinicopathological parameters. BMC Cancer.

[114] Castel D, Philippe C, Calmon R, Le Dret L, Truffaux N, Boddaert N (2015). Histone H3F3A and HIST1H3B K27M mutations define two subgroups of diffuse intrinsic pontine gliomas with different prognosis and phenotypes. Acta Neuropathol.

[115] Gillison ML, Koch WM, Capone RB, Spafford M, Westra WH, Wu L (2000). Evidence for a causal association between human papillomavirus and a subset of head and neck cancers. J Natl Cancer Inst.

[116] Munoz N, Bosch FX, de Sanjose S, Herrero R, Castellsague X, Shah KV (2003). Epidemiologic classification of human papillomavirus types associated with cervical cancer. N Engl J Med.

[117] Frisch M, Glimelius B, van den Brule AJ, Wohlfahrt J, Meijer CJ, Walboomers JM (1997). Sexually transmitted infection as a cause of anal cancer. N Engl J Med.

[118] Kozakova L, Vondrova L, Stejskal K, Charalabous P, Kolesar P, Lehmann AR (2015). The melanoma-associated antigen 1 (MAGEA1) protein stimulates the E3 ubiquitin-ligase activity of TRIM31 within a TRIM31-MAGEA1-NSE4 complex. Cell Cycle.

[119] Mao Y, Fan W, Hu H, Zhang L, Michel J, Wu Y (2019). MAGE-A1 in lung adenocarcinoma as a promising target of chimeric antigen receptor T cells. J Hematol Oncol.

[120] Newman JA, Cooper CD, Roos AK, Aitkenhead H, Oppermann UC, Cho HJ (2016). Structures of two melanoma-associated antigens suggest allosteric regulation of effector binding. PLoS ONE.

[121] Boel P, Wildmann C, Sensi ML, Brasseur R, Renauld JC, Coulie P (1995). BAGE: a new gene encoding an antigen recognized on human melanomas by cytolytic T lymphocytes. Immunity.

[122] De Plaen E, Arden K, Traversari C, Gaforio JJ, Szikora JP, De Smet C (1994). Structure, chromosomal localization, and expression of 12 genes of the MAGE family. Immunogenetics.

[123] Hagiwara Y, Sieverling L, Hanif F, Anton J, Dickinson ER, Bui TT (2016). Consequences of point mutations in melanoma-associated antigen 4 (MAGE-A4) protein: insights from structural and biophysical studies. Sci Rep.

[124] Fujiwara-Kuroda A, Kato T, Abiko T, Tsuchikawa T, Kyogoku N, Ichinokawa M (2018). Prognostic value of MAGEA4 in primary lung cancer depends on subcellular localization and p53 status. Int J Oncol.

[125] Bergeron A, Picard V, LaRue H, Harel F, Hovington H, Lacombe L (2009). High frequency of MAGE-A4 and MAGE-A9 expression in high-risk bladder cancer. Int J Cancer.

[126] Doyle JM, Gao J, Wang J, Yang M, Potts PR (2010). MAGE-RING protein complexes comprise a family of E3 ubiquitin ligases. Mol Cell.

[127] Ayyoub M, Scarlata CM, Hamai A, Pignon P, Valmori D (2014). Expression of MAGE-A3/6 in primary breast cancer is associated with hormone receptor negative status, high histologic grade, and poor survival. J Immunother.

[128] Endo M, Kanda M, Sawaki K, Shimizu D, Tanaka C, Kobayashi D (2019). Tissue expression of melanoma-associated antigen A6 and clinical characteristics of gastric cancer. Anticancer Res.

[129] Fon Tacer K, Montoya MC, Oatley MJ, Lord T, Oatley JM, Klein J (2019). MAGE cancer-testis antigens protect the mammalian germline under environmental stress. Sci Adv.

[130] Huang LQ, Brasseur F, Serrano A, De Plaen E, van der Bruggen P, Boon T (1999). Cytolytic T lymphocytes recognize an antigen encoded by MAGE-A10 on a human melanoma. J Immunol.

[131] Bar-Haim E, Paz A, Machlenkin A, Hazzan D, Tirosh B, Carmon L (2004). MAGE-A8 overexpression in transitional cell carcinoma of the bladder: identification of two tumour-associated antigen peptides. Br J Cancer.

[132] Schultz-Thater E, Piscuoglio S, Iezzi G, Le Magnen C, Zajac P, Carafa V (2011). MAGE-A10 is a nuclear protein frequently expressed in high percentages of tumor cells in lung, skin and urothelial malignancies. Int J Cancer.

[133] Spurgeon ME, Lambert PF (2013). Merkel cell polyomavirus: a newly discovered human virus with oncogenic potential. Virology.

[134] Chang K, Pastan I (1996). Molecular cloning of mesothelin, a differentiation antigen present on mesothelium, mesotheliomas, and ovarian cancers. Proc Natl Acad Sci USA.

[135] Inaguma S, Wang Z, Lasota J, Onda M, Czapiewski P, Langfort R (2017). Comprehensive immunohistochemical study of mesothelin (MSLN) using different monoclonal antibodies 5B2 and MN-1 in 1562 tumors with evaluation of its prognostic value in malignant pleural mesothelioma. Oncotarget.

[136] Madeira K, Dondossola ER, Farias BF, Simon CS, Alexandre MC, Silva BR (2016). Mesothelin as a biomarker for ovarian carcinoma: a meta-analysis. An Acad Bras Cienc.

[137] Le K, Wang J, Zhang T, Guo Y, Chang H, Wang S (2020). Overexpression of mesothelin in pancreatic ductal adenocarcinoma (PDAC). Int J Med Sci.

[138] Simpson AJ, Caballero OL, Jungbluth A, Chen YT, Old LJ (2005). Cancer/testis antigens, gametogenesis and cancer. Nat Rev Cancer.

[139] Aung PP, Liu YC, Ballester LY, Robbins PF, Rosenberg SA, Lee CC (2014). Expression of New York esophageal squamous cell carcinoma-1 in primary and metastatic melanoma. Hum Pathol.

[140] Sugita Y, Wada H, Fujita S, Nakata T, Sato S, Noguchi Y (2004). NY-ESO-1 expression and immunogenicity in malignant and benign breast tumors. Cancer Res.

[141] Szender JB, Papanicolau-Sengos A, Eng KH, Miliotto AJ, Lugade AA, Gnjatic S (2017). NY-ESO-1 expression predicts an aggressive phenotype of ovarian cancer. Gynecol Oncol.

[142] Lee L, Wang RF, Wang X, Mixon A, Johnson BE, Rosenberg SA (1999). NY-ESO-1 may be a potential target for lung cancer immunotherapy. Cancer J Sci Am.

[143] Ikeda H, Lethe B, Lehmann F, van Baren N, Baurain JF, de Smet C (1997). Characterization of an antigen that is recognized on a melanoma showing partial HLA loss by CTL expressing an NK inhibitory receptor. Immunity.

[144] Szczepanski MJ, Whiteside TL (2013). Elevated PRAME expression: what does this mean for treatment of head and neck squamous cell carcinoma?. Biomark Med.

[145] Tan P, Zou C, Yong B, Han J, Zhang L, Su Q (2012). Expression and prognostic relevance of PRAME in primary osteosarcoma. Biochem Biophys Res Commun.

[146] Buckler AJ, Pelletier J, Haber DA, Glaser T, Housman DE (1991). Isolation, characterization, and expression of the murine Wilms' tumor gene (WT1) during kidney development. Mol Cell Biol.

[147] Campbell CE, Kuriyan NP, Rackley RR, Caulfield MJ, Tubbs R, Finke J (1998). Constitutive expression of the Wilms tumor suppressor gene (WT1) in renal cell carcinoma. Int J Cancer.

[148] Miyoshi Y, Ando A, Egawa C, Taguchi T, Tamaki Y, Tamaki H (2002). High expression of Wilms' tumor suppressor gene predicts poor prognosis in breast cancer patients. Clin Cancer Res.

[149] Miwa H, Beran M, Saunders GF (1992). Expression of the Wilms' tumor gene (WT1) in human leukemias. Leukemia.

[150] Stromnes IM, Schmitt TM, Chapuis AG, Hingorani SR, Greenberg PD (2014). Re-adapting T cells for cancer therapy: from mouse models to clinical trials. Immunol Rev.

[151] Linnemann C, Mezzadra R, Schumacher TN (2014). TCR repertoires of intratumoral T-cell subsets. Immunol Rev.

[152] Pardoll DM (2012). The blockade of immune checkpoints in cancer immunotherapy. Nat Rev Cancer.

[153] Siska PJ, Rathmell JC (2015). T cell metabolic fitness in antitumor immunity. Trends Immunol.

[154] John LB, Devaud C, Duong CP, Yong CS, Beavis PA, Haynes NM (2013). Anti-PD-1 antibody therapy potently enhances the eradication of established tumors by gene-modified T cells. Clin Cancer Res.

[155] Chowell D, Morris LGT, Grigg CM, Weber JK, Samstein RM, Makarov V (2018). Patient HLA class I genotype influences cancer response to checkpoint blockade immunotherapy. Science.

[156] Magalhaes I, Carvalho-Queiroz C, Hartana CA, Kaiser A, Lukic A, Mints M (2019). Facing the future: challenges and opportunities in adoptive T cell therapy in cancer. Expert Opin Biol Ther.

[157] Stadtmauer EA, Fraietta JA, Davis MM, Cohen AD, Weber KL, Lancaster E (2020). CRISPR-engineered T cells in patients with refractory cancer. Science.

